# Active Matter, Microreversibility, and Thermodynamics

**DOI:** 10.34133/2020/9739231

**Published:** 2020-05-21

**Authors:** Pierre Gaspard, Raymond Kapral

**Affiliations:** ^1^Center for Nonlinear Phenomena and Complex Systems, Université Libre de Bruxelles (U.L.B.), Code Postal 231, Campus Plaine, B-1050 Brussels, Belgium; ^2^Chemical Physics Theory Group, Department of Chemistry, University of Toronto, Toronto, Ontario, Canada M5S 3H6

## Abstract

Active matter, comprising many active agents interacting and moving in fluids or more complex environments, is a commonly occurring state of matter in biological and physical systems. By its very nature, active matter systems exist in nonequilibrium states. In this paper, the active agents are small Janus colloidal particles that use chemical energy provided by chemical reactions occurring on their surfaces for propulsion through a diffusiophoretic mechanism. As a result of interactions among these colloids, either directly or through fluid velocity and concentration fields, they may act collectively to form structures such as dynamic clusters. A general nonequilibrium thermodynamics framework for the description of such systems is presented that accounts for both self-diffusiophoresis and diffusiophoresis due to external concentration gradients, and is consistent with microreversibility. It predicts the existence of a reciprocal effect of diffusiophoresis back onto the reaction rate for the entire collection of colloids in the system, as well as the existence of a clustering instability that leads to nonequilibrium inhomogeneous system states.

## 1. Introduction

Active matter is composed of motile entities or agents interacting with each other either directly or through the velocity and concentration fields of the medium in which they move. Such interactions lead to collective dynamics giving rise to states of matter that may differ from those in equilibrium systems. The study of such collective behavior presents challenges and is currently a topic of considerable scientific interest. Systems with many complex agents can be investigated in different ways. One way is to describe collective dynamics at the macroscale in terms of fields representing the distribution of the agents across the system. These fields are ruled by partial differential equations that are established using general symmetries and experimental observations. Another approach is to model active matter as being composed of active particles moving in space according to specific rules that are postulated on the basis of empirical considerations.

Both of these approaches have been used to explore the origins and types of collective dynamics that can be found in active matter systems, and research on this topic ranges from studies of simple active particle models, often satisfying minimal rules, to suspensions of more complex active synthetic or biological agents [1–11]. The collective behavior in systems where the active agents are chemically propelled colloids, the subject of this paper, has also been the topic of experimental and theoretical research [12–25].

Systems containing colloidal particles are governed by physicochemical laws, so that their time evolution can be understood from first principles using statistical-mechanical methods. This approach was pioneered by Einstein [26] and Smoluchowski [27–29] at the beginning of the twentieth century and systematically developed since then for passive colloidal particles [30–33]. In active matter, the colloidal particles are propelled with energy supplied by the surrounding solution, so that the description should be extended to include the molecular concentrations of fuel and product powering their motion, in addition to the velocity field of the fluid. Through such an approach, active matter can be described from the scale of a single colloidal motor moving in the surrounding fluid, up to the macroscale where many colloidal motors generate collective motion by interaction. At the macroscale, collective dynamics is described in terms of the distribution function giving the orientation as well as the position of the colloidal motors. This statistical-mechanical approach has the advantage that the parameters characterizing active matter at the macroscale can be deduced from the microscopic level of description. The knowledge of these parameters in terms of the properties of materials composing the colloidal motors and the surrounding solution is fundamental for engineering active systems.

The present paper contributes to the statistical-mechanical and nonequilibrium thermodynamic approaches for active matter systems [34–42], and considers systems whose active agents are Janus colloids with catalytic and noncatalytic faces moving by diffusiophoresis generated by chemical reactions taking place on their catalytic faces or caps [43, 44, 40]. Because of diffusiophoresis, the velocity and concentration fields are coupled together in the fluid around the Janus particle [45]. We start from the calculation of the diffusiophoretic force and torque on a single Janus particle moving in a fluid in the presence of molecular species corresponding to the fuel and the product of the reaction taking place on its catalytic surface. The concentrations of these molecular species may develop gradients on large scales under nonequilibrium conditions, and these gradients should be included in the calculation of the force and torque. The resulting diffusiophoretic force and torque enter the coupled Langevin equations ruling the displacement, rotation, and overall reaction of a single active particle.

Next, the evolution equation is established for the distribution function of the ensemble of active particles in a dilute colloidal solution. In order to be consistent with microreversibility, the principles of nonequilibrium thermodynamics are used to relate the thermodynamic forces or affinities to the current densities with linear response coefficients satisfying Onsager's reciprocal relations [46–53]. This method allows us to obtain all the possible couplings compatible with microreversibility, including *a priori* unexpected reciprocal effects. Moreover, this method provides an expression for the entropy production rate density for active matter in agreement with the second law of thermodynamics and including the contribution of the reaction powering activity. Through this procedure, macroscopic evolution equations are obtained that govern the collective dynamics of colloidal motors coupled to the molecular concentrations of fuel and product. These equations can be shown to generate the reciprocal effect of diffusiophoresis back onto the reaction rate that has been obtained previously for a single particle [39, 40], but now at the macroscale. Furthermore, pattern formation due to a clustering instability manifests itself under nonequilibrium conditions induced by a bulk reaction replenishing the solution with fuel.

The paper is organized as follows.  Section 2 is devoted to the dynamics of a single colloidal motor. The force and torque due to diffusiophoresis are deduced by solving the diffusion equations for the molecular concentrations coupled to the Navier-Stokes equations for the fluid velocity, including the contributions of concentration gradients at large distances from the particle. These contributions were neglected previously [39, 40] and are calculated in detail here. In Section 3, the diffusiophoretic force and torque obtained in Section 2 are incorporated into the evolution equation for the distribution function describing the ensemble of colloidal motors, and the entropy production rate density is explicitly obtained. Two implications of these results are presented in Sections 4 and 5. First, the reciprocal effect due to the diffusiophoretic coupling of an external force and torque back onto the reaction rate is recovered, now at the level of the collective dynamics. Second, a clustering instability leading to pattern formation is shown to manifest itself. The conclusions of the research are given in Section 6. The appendices provide additional details of the calculations.

## 2. Diffusiophoresis and Colloidal Motors

**Figure 1:**
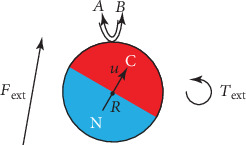
Schematic representation of a Janus particle with its catalytic (C) and noncatalytic (N) hemispheres where the surface reaction (1) takes place between fuel *A* and product *B* supplied by the solution surrounding the particle. The particle is also subjected to some external force *F*_ext_ and torque *T*_ext_. The position of its center of mass is *R*, and *u* is the unit vector giving its orientation and pointing in the direction of the catalytic hemisphere.

This section describes the motion of a single spherical Janus colloidal motor of radius *R* that is propelled by self-diffusiophoresis generated by a reversible reaction,

(1) $$\begin{array}{c} A+C\overset{\kappa_{+}}{\underset{\kappa_{-}}{⇄}}B+C, \end{array}$$

with rate constants *κ*_±_ taking place on its catalytic surface, as depicted in Figure 1. In this reaction, *A* is the fuel and *B* the product, which are present in the solution surrounding the particle. Moreover, the concentrations of the *A* and *B* molecular species are assumed to have gradients *g*_*k*_ with *k* = *A*, *B* at large distances from the particle that also contribute to motion by diffusiophoresis; thus, the motion of the particle is determined by processes in the fluid surrounding the particle.

### 2.1. Chemohydrodynamics around a Colloidal Motor

In order to determine the force and the torque due to diffusiophoresis, as well as the overall reaction rate, the velocity of the fluid and the concentrations of fuel *A* and product *B* should be obtained by solving the Navier-Stokes equations for the fluid velocity *v* = *v*_fluid_ coupled to the advection-diffusion equations for the molecular concentrations *c*_*k*_ with *k* = *A*, *B*:

(2) $$\begin{array}{c} \rho \left(\partial_{t}v+v\cdot \nabla v\right)=-\nabla p+\eta \nabla^{2}v, \end{array}$$

(3) $$\begin{array}{c} \nabla \cdot v=0, \end{array}$$

(4) $$\begin{array}{c} \partial_{t}c_{k}+v\cdot \nabla c_{k}=D_{k}\nabla^{2}c_{k}, \end{array}$$

where *ρ* is the constant mass density (the fluid being assumed to be incompressible), *p* the hydrostatic pressure, *η* the shear viscosity, and *D*_*k*_ the molecular diffusivity of species *k*.

The coupling between the velocity and concentration fields is established with the boundary conditions [40, 54]

(5) $$\begin{array}{c} n\cdot {\left(v-v_{\text{solid}}\right)}_{R}=0, \end{array}$$

(6) $$\begin{array}{c} 1_{⊥}\cdot {\left(v-v_{\text{solid}}\right)}_{R}=1_{⊥}\cdot {\left[b{\left(\nabla v\right)}^{S}-\underset{k}{\sum }\;b_{k}\nabla c_{k}\right]}_{R}, \end{array}$$

(7) $$\begin{array}{c} D_{k}{\left(n\cdot \nabla c_{k}\right)}_{R}=-\nu_{k}{\left(\kappa_{+}c_{A}-\kappa_{-}c_{B}\right)}_{R}, \end{array}$$

where *n* is the unit vector normal to the solid surface, 1_⊥_ ≡ 1 − *nn*, *b* is the slip length, (∇*v*)^*S*^ = (∇*v*+∇*v*^*T*^), *T* denotes the transpose, *b*_*k*_ is the diffusiophoretic coefficient of species *k* coupling the velocity field to the corresponding concentration field because of different interactions between the solid surface with the molecules of different species. The velocity field inside the solid particle is given by *v*_solid_ = *V* + **Ω** × (*r* − *R*) in terms of the translational and angular velocities of the particle, respectively, denoted by *V* and **Ω**, and position *R* of the center of mass of the particle. Equations (5) and (6) are the boundary conditions on the components of the velocity field that are, respectively, normal and tangential to the interface *Σ*(*t*), which is located on the sphere ||*r* − *R*|| = *R*. The last equation, i.e., equation (7) is the boundary condition for the two reacting species *k* = *A*, *B*, where *ν*_*k*_ is the stoichiometric coefficient of species *k* in the reaction (*ν*_*A*_ = −1 and *ν*_*B*_ = +1), and *κ*_±_ are the forward and reverse surface rate constants per unit area.

The velocity field is assumed to vanish at large distances from the particle, so that the fluid is at rest except in the vicinity of the colloid. With the aim of obtaining mean-field equations for a dilute suspension of active particles, we also assume that the concentration fields can have nonvanishing gradients on large spatial scales. Accordingly, the concentration gradients (∇*c*_*k*_)_∞_ = *g*_*k*_ are taken to exist at large distances from the colloidal particle.

We suppose that the diffusiophoretic coefficients take the values *b*_*k*_^*c*^ and *b*_*k*_^*n*^ on the catalytic and noncatalytic hemispheres, respectively, while the surface rate constants per unit area take positive values *κ*_±_^*c*^ on the catalytic hemisphere and vanish on the noncatalytic hemisphere, *κ*_±_^*n*^ = 0. Using spherical coordinates (*θ*, *φ*) with polar angle *θ* defined with respect to the axis of the cylindrical symmetry of the Janus particle, we have

(8) $$\begin{array}{c} b_{k}\left(\theta ,\varphi \right)=\underset{h=c,n}{\sum }\;b_{k}^{h}H^{h}\left(\theta \right),\\ \kappa_{\pm }\left(\theta ,\varphi \right)=\underset{h=c,n}{\sum }\;\kappa_{\pm }^{h}H^{h}\left(\theta \right), \end{array}$$

where *H*^*h*^(*θ*) denotes the Heaviside function such that *H*^*h*^(*θ*) = 1 on hemisphere *h* and is zero otherwise. The catalytic hemisphere is taken as 0 ≤ *θ* ≤ (*π*/2), and the noncatalytic hemisphere as (*π*/2) < *θ* ≤ *π*.

Solving equations (2)–(4) with the boundary conditions (5)–(7), the velocity and concentration fields can be obtained in the vicinity of the colloidal motor [45, 40, 41]. Accordingly, the force and the torque exerted by the fluid on the motor, as well as the overall reaction rate at its catalytic surface, are given by the following surface integrals at the fluid-colloid interface *Σ*(*t*),

(9) $$\begin{array}{c} F=-\int_{\sum \left(t\right)}\text{ P}\cdot n\;d\Sigma ,\\ T=-\int_{\sum \left(t\right)}\;\left(r-R\right)\times \left(\text{P}\cdot n\right)d\Sigma ,\\ W=\int_{\sum \left(t\right)}\;\left(\kappa_{+}\;c_{A}-\kappa_{-}c_{B}\right)d\Sigma , \end{array}$$

where P = *p*1 − *η*(∇*v*)^*S*^ is the pressure tensor of the fluid. The fluctuating contributions from thermal noise can also be included [40, 41].

### 2.2. Coupled Langevin Equations for the Motor

The orientation of the Janus particle is described by the unit vector *u* attached to the axis of the cylindrical symmetry of the Janus particle and pointing towards the catalytic hemisphere. Accordingly, the displacement and rotation of the particle are ruled by

(10) $$\begin{array}{c} \frac{dR}{dt}=V,\\ \frac{du}{dt}=\Omega \times u, \end{array}$$

in terms of the translational and rotational velocities. These velocities, as well as the number *N* of reactive events taking place on the particle, are governed by the following coupled Langevin equations [39, 40, 41]:

(11) $$\begin{array}{c} M\frac{dV}{dt}=-\gamma_{t}V+F_{d}+F_{\text{ext}}+F_{fl}\left(t\right), \end{array}$$

(12) $$\begin{array}{c} I\cdot \frac{d\Omega }{dt}=-\gamma_{r}\Omega +T_{d}+T_{\text{ext}}+T_{fl}\left(t\right), \end{array}$$

(13) $$\begin{array}{c} \frac{dN}{dt}=W_{rxn}+W_{d}+W_{fl}\left(t\right), \end{array}$$

where *M* and *I* denote the mass and inertia tensor of the motor, *γ*_*t*_ = 6*πηR*(1 + (2*b*/*R*))/(1 + (3*b*/*R*)) is the translational friction coefficient, *γ*_*r*_ = 8*πηR*^3^/(1 + (3*b*/*R*)) the rotational friction coefficient, *F*_*d*_ and *T*_*d*_ the diffusiophoretic force and torque, *F*_ext_ and *T*_ext_ the external force and torque exerted on the particle, while *F*_*fl*_(*t*) and *T*_*fl*_(*t*) are the contributions to the force and torque due to thermal fluctuations. The overall net reaction rate is *W*_*rxn*_, *W*_*d*_ is the reciprocal contribution of diffusiophoresis back onto the reaction rate, and *W*_*fl*_(*t*) is the fluctuating reaction rate. If the Janus particle has a magnetic dipole *μ* and is subjected to an external magnetic field *B*, then the external torque would be given by *T*_ext_ = *μu* × *B*. In the overdamped regime, the coupled Langevin equations are obtained by neglecting the inertial terms in equations (11) and (12).

Solving the Navier-Stokes equations (2) and (3) coupled to equation (4) for molecular concentrations with the boundary conditions (5)–(7), the force and the torque exerted on a spherical particle of radius *R* in a fluid with shear viscosity *η* and the overall net reaction rate are given by [40]

(14) $$\begin{array}{c} F_{d}=\frac{6\pi \eta R}{1+\left(3b/R\right)}\underset{k}{\sum }{\overset{¯}{b_{k}1_{⊥}\cdot \nabla c_{k}}}^{s}, \end{array}$$

(15) $$\begin{array}{c} T_{d}=\frac{12\pi \eta R}{1+\left(3b/R\right)}\underset{k}{\sum }{\overset{¯}{b_{k}r\times \nabla c_{k}}}^{s}, \end{array}$$

(16) $$\begin{array}{c} W_{rxn}=4\pi R^{2}{\overset{¯}{\kappa_{+}c_{A}-\kappa_{-}c_{B}}}^{s}, \end{array}$$

expressed in terms of the surface average

(17) $$\begin{array}{c} {\overset{¯}{\left(\cdot \right)}}^{s}=\frac{1}{4\pi }\int {\left(\cdot \right)}_{r=R}d\cos\theta d\varphi . \end{array}$$

The expressions (14) and (15) find their origin in the generalization of Faxén's theorem to a sphere moving in a time-dependent velocity field [55, 56]. When writing these equations, we have taken into account the possibility that the diffusiophoretic coefficients *b*_*k*_ and the surface rate constants *κ*_±_ may be nonuniform on the particle surface.

### 2.3. Motion in Molecular Concentration Gradients

If molecular diffusion is fast enough so that the concentration fields adopt stationary profiles around the catalytic particle in the concentration gradients *g*_*k*_, the diffusiophoretic translational and rotational velocities can be written as follows (see Appendix A):

(18) $$\begin{array}{c} V_{d}=\frac{F_{d}}{\gamma_{t}}=V_{\text{s}d}u+\underset{k}{\sum }\left(\xi_{k}1+\varepsilon_{k}Q_{u}\right)\cdot g_{k}, \end{array}$$

(19) $$\begin{array}{c} \Omega_{d}=\frac{T_{d}}{\gamma_{r}}=\underset{k}{\sum }\lambda_{k}u\times g_{k}, \end{array}$$

where the parameters *ξ*_*k*_, *ε*_*k*_, and *λ*_*k*_ are given in equations (A.35)–(A.40) in terms of the diffusiophoretic coefficients *b*_*k*_^*h*^, the rate constants per unit area *κ*_±_^*c*^, the slip length *b*, the molecular diffusivities *D*_*k*_, and the geometry of the Janus particle. The 3 × 3 identity matrix is 1, while

(20) $$\begin{array}{c} Q_{u}\equiv uu-\frac{1}{3}1. \end{array}$$

The self-diffusiophoretic velocity, expressed in terms of the molecular concentrations ${\bar{c}}_{k}$ extrapolated to the center of the particle, is

(21) $$\begin{array}{c} V_{\text{s}d}=\underset{k}{\sum }\zeta_{k}{\bar{c}}_{k}=\varsigma \left(\kappa_{+}^{c}{\bar{c}}_{A}-\kappa_{-}^{c}{\bar{c}}_{B}\right), \end{array}$$

since the parameters *ζ*_*k*_ may be written in the forms *ζ*_*A*_ = *ςκ*_+_^*c*^ and *ζ*_*B*_ = −*ςκ*_−_^*c*^ (see Appendix A, equations (A.33)-(A.34).

In the absence of a reaction, we recover the diffusiophoretic velocities given in Refs. [57, 58]:

(22) $$\begin{array}{c} V_{d}=\underset{k}{\sum }\xi_{k0}g_{k}\;\text{with }\xi_{k0}=\frac{b_{k}^{c}+b_{k}^{n}}{2\left(1+\left(2b/R\right)\right)},\\ \Omega_{d}=\underset{k}{\sum }\lambda_{k0}u\times g_{k}\;\text{with }\lambda_{k0}=\frac{9}{16R}\left(b_{k}^{c}-b_{k}^{n}\right). \end{array}$$

Moreover, if the diffusiophoretic coefficients are the same on both hemispheres *b*_*k*_^*c*^ = *b*_*k*_^*n*^, the angular velocity is equal to zero, **Ω**_*d*_ = 0.

In the presence of a reaction, but without gradients (*g*_*k*_ = 0), we have $\kappa_{+}^{c}{\bar{c}}_{A}\neq \kappa_{-}^{c}{\bar{c}}_{B}$ and the linear velocity reduces to the contribution of self-diffusiophoresis, *V*_*d*_ = *V*_*sd*_*u*, characterizing the activity of the Janus particle.

The overall reaction rate can be written as follows:

(23) $$\begin{array}{c} W_{rxn}=k_{+}{\bar{c}}_{A}-k_{-}{\bar{c}}_{B}+\varpi \;\left(k_{+}g_{A}-k_{-}g_{B}\right)\cdot u, \end{array}$$

in terms of rate constants *k*_±_ = Γ*κ*_±_^c^ and a parameter *ϖ* = *O*(*R*) given in equation (A.45). In the absence of the concentration gradients, we recover the expression obtained in Ref. [40]. In the presence of the concentration gradients *g*_*k*_, there is an extra contribution depending on the direction *u* of the Janus particle. However, this last term is normally negligible because we typically have $R||g_{k}||\ll {\bar{c}}_{k}$ for micrometric particles and macroscopic gradients of molecular concentrations.

We note that both the self-diffusiophoretic velocity (21) and the leading term of the reaction rate (23) are proportional to each other. Their ratio defines the self-diffusiophoretic parameter *χ* which was introduced in Refs. [39, 40],

(24) $$\begin{array}{c} \chi \equiv \frac{V_{\text{s}d}}{k_{+}{\bar{c}}_{A}-k_{-}{\bar{c}}_{B}}=\frac{\varsigma }{\Gamma }, \end{array}$$

where the last equality was obtained using *k*_±_ = Γ*κ*_±_^c^.

## 3. Active Suspension of Colloidal Motors

### 3.1. Onsager's Reciprocal Relations

We now show that Onsager's principle of nonequilibrium thermodynamics [46–53] can be used to establish coupled diffusion-reaction equations of motion for active matter that are consistent with microreversibility. According to Onsager's principle, currents are related to thermodynamic forces (or affinities) by

(25) $$\begin{array}{c} J_{\alpha }=\underset{\beta }{\sum }L_{\alpha \beta }A^{\beta }, \end{array}$$

where the linear response coefficients satisfy the Onsager reciprocal relations,

(26) $$\begin{array}{c} L_{\alpha \beta }=L_{\beta \alpha }, \end{array}$$

if the affinities are even under time reversal. The thermodynamic entropy production rate density is given by

(27) $$\begin{array}{c} \sigma_{s}=k_{\text{B}}\underset{\alpha }{\sum }J_{\alpha }A^{\alpha }=k_{\text{B}}\underset{\alpha \beta }{\sum }L_{\alpha \beta }A^{\alpha }A^{\beta }\geq 0, \end{array}$$

where *k*_B_ is Boltzmann's constant.

### 3.2. Mean-Field Equations for the Active Suspension

The system we consider is a dilute solution containing the reactive molecular *A* and *B* species together with colloidal motors *C* in an inert solvent *S*. The motors are spherical Janus particles and, as described in Section 2, have hemispherical catalytic surfaces where the reaction *A*⇄*B* takes place. Moreover, we suppose that the solution is globally at rest, so that the velocity field is equal to zero on scales larger than the size of colloids. The solution is described at the macroscale in terms of the molecular densities *n*_*A*_(*r*, *t*) and *n*_*B*_(*r*, *t*), as well as the distribution function of the colloidal motors, *f*(*r*, *u*, *t*), where *r* = (*x*, *y*, *z*) is the position and *u* = (sin*θ*cos*φ*, sin*θ*sin*φ*, cos*θ*) is the unit vector giving the orientation of the Janus particles (expressed in spherical coordinates in the laboratory frame). The distribution function is defined as

(28) $$\begin{array}{c} f\left(r,u,t\right)\equiv \overset{N_{C}}{\underset{i=1}{\sum }}\;\delta^{3}\left[r-r_{i}\left(t\right)\right]\delta^{2}\left[u-u_{i}\left(t\right)\right], \end{array}$$

where {*r*_*i*_, *u*_*i*_}_*i*=1_^*N*_*C*_^ are the positions and orientational unit vectors of the colloidal motors. For a dilute suspension, the evolution equation of this distribution function can be deduced from the Fokker-Planck equation for the probability that a single colloidal motor is located at the position *r* with the orientation *u* [39, 40, 41]. Once, this distribution function is known, we can obtain the successive moments of *u*:

(29) $$\begin{array}{c} n_{C}\left(r,t\right)\equiv \int f\left(r,u,t\right)d^{2}u, \end{array}$$

(30) $$\begin{array}{c} p\left(r,t\right)\equiv \int uf\left(r,u,t\right)d^{2}u, \end{array}$$

(31) $$\begin{array}{c} \begin{array}{c} q\left(r,t\right)\equiv \int Q_{u}f\left(r,u,t\right)d^{2}u,\\ \vdots \end{array} \end{array}$$

where *d*^2^*u* = *d*cos*θdφ*, *n*_*C*_ is the density or concentration of colloidal motors, *p* is the polar order parameter or polarization of the colloidal motors, and *q* is the traceless order parameter analogous to that for apolar nematic liquid crystals expressed in terms of the tensor (20) and, thus, satisfies *tr*(*q*) = 0.

At the macroscale, the reaction is

(32) $$\begin{array}{c} A+C\overset{k_{+}}{\underset{k_{-}}{⇄}}B+C, \end{array}$$

with the rate constants *k*_±_. For the colloidal suspension treated here, the reaction should be described by a reaction rate density *w* that is proportional to the distribution function of colloidal motors and determined by the surface reaction taken into account with the boundary conditions (7) in Section 2.

The mean concentrations of molecular species are defined by $n_{k}=\left(1-\phi \right){\bar{c}}_{k}$, where *ϕ* = 4*πR*^3^*n*_*C*_/3 is the volume fraction of the suspension. Their corresponding gradients are related to those considered in Section 2 by ∇*n*_*k*_ = (1 − *ϕ*)*g*_*k*_ for a dilute enough suspension. The coupled diffusion-reaction equations for the different species take the following forms:

(33) $$\begin{array}{c} \partial_{t}n_{k}+\nabla \cdot j_{k}=\nu_{k}w\;\left(k=A,B\right), \end{array}$$

(34) $$\begin{array}{c} \partial_{t}f+\nabla \cdot \left(Vf-D_{t}\nabla f\right)=D_{r}L_{r}f, \end{array}$$

where *j*_*k*_ are the molecular current densities, *V* is the total drift velocity obtained by adding the drift velocity due to the external force *V*_ext_ = *F*_ext_/*γ*_*t*_ to the diffusiophoretic velocity (18) giving

(35) $$\begin{array}{c} V=V_{\text{s}d}u+\underset{k}{\sum }\;\left(\xi_{k}\;1+\varepsilon_{k}{\text{Q}}_{u}\right)\cdot \nabla n_{k}+\beta D_{t}F_{\text{ext}}, \end{array}$$

with the self-diffusiophoretic velocity (equation (21))

(36) $$\begin{array}{c} V_{\text{s}d}=\underset{k}{\sum }\zeta_{k}n_{k}=\varsigma \left(\kappa_{+}^{\text{c}}n_{A}-\kappa_{-}^{\text{c}}n_{B}\right), \end{array}$$

now expressed in terms of the mean concentrations *n*_*k*_, and the inverse temperature *β* = (*k*_B_*T*)^−1^. In equation (34), *D*_*t*_ is an effective translational diffusion coefficient related to the effective translational friction coefficient by Einstein's formula *D*_*t*_ = *k*_B_*T*/*γ*_*t*_ and *D*_*r*_ is an effective rotational diffusion coefficient related to the effective rotational friction coefficient by *D*_*r*_ = *k*_B_*T*/*γ*_*r*_. Since the shear viscosity increases as *η*≃*η*^(0)^(1 + 2.5*ϕ*) with the volume fraction *ϕ* of the suspension [26, 31], both friction coefficients *γ*_*t*_ and *γ*_*r*_ also increase, and the diffusion coefficients decrease. In particular, it is known that *D*_*t*_≃*D*_*t*_^(0)^(1 − 2.1*ϕ*) [31]. A similar dependence on the volume fraction *ϕ* is expected for the parameters *ς*, *ξ*_*k*_, *ε*_*k*_, and *λ*_*k*_ given in Appendix A, since these parameters are proportional to the diffusiophoretic coefficients *b*_*k*_^*h*^ that are known to be inversely proportional to shear viscosity, *b*_*k*_^*h*^ ∝ *η*^−1^ [54, 57, 58]. The effects of this dependence would manifest themselves if the colloidal suspension became dense enough. Here, such effects are assumed to play a negligible role.

The Janus particles have a spherical shape so that their random rotational and translational motions are decoupled. In this case, the rotational diffusion operator is given by

(37) $$\begin{array}{c} L_{r}f=\frac{1}{\sin\theta }\;\partial_{\theta }\left[\sin\theta {\text{e}}^{-\beta U_{r}}\partial_{\theta }\left({\text{e}}^{\beta U_{r}}f\right)\right]+\frac{1}{\sin^{2}\theta }\partial_{\varphi }\left[{\text{e}}^{-\beta U_{r}}\partial_{\varphi }\left({\text{e}}^{\beta U_{r}}f\right)\right], \end{array}$$

expressed in terms of the rotational energy associated with the torque exerted by an external magnetic field *B* on some magnetic dipole *μ* of the particle [52] and that due to the diffusiophoretic effect, we have the following:

(38) $$\begin{array}{c} U_{r}=-\mu B\cdot u-\gamma_{r}\left(\underset{k}{\sum }\lambda_{k}\nabla n_{k}\right)\cdot u. \end{array}$$

### 3.3. Translational and Rotational Current Densities

Before proceeding with nonequilibrium thermodynamics, we need to identify in equation (34) the current densities associated with the translational and rotational movements of the colloidal motors. The distribution function *f*(*r*, *u*) for colloidal Janus particles is defined in the five-dimensional space (*x*, *y*, *z*, *θ*, *φ*), where (*x*, *y*, *z*) are the Cartesian coordinates for the position *r* and (*θ*, *φ*) the spherical coordinates for the orientation *u*. Vector calculus is used in these coordinates to obtain the corresponding gradients and divergences [59].

For the rotational degrees of freedom we have

(39) $$\begin{array}{c} du^{2}=d\theta^{2}+\sin^{2}\theta d\varphi^{2}=g_{ij}dq^{i}dq^{j}\;\text{with }\left(g_{ij}\right)=\left(\begin{array}{cc} 1 & 0\\ 0 & \sin^{2}\theta \end{array}\right). \end{array}$$

The scalar product between a pair of rotational vectors *a*_*r*_, *b*_*r*_ ∈ ℝ^2^ is given by *a*_*r*_ · *b*_*r*_ = ∑_*i*,*j*=*θ*,*φ*_*g*_*ij*_*a*_*r*_^*i*^*b*_*r*_^*j*^, and the scalar product of such a vector with itself is denoted *a*_*r*_^2^ = *a*_*r*_ · *a*_*r*_. In spherical coordinates, the rotational gradient and divergence are given, respectively, by [59]

(40) $$\begin{array}{c} {\mathrm{grad}}_{r}X=\left(\begin{array}{c} \partial_{\theta }X\\ \frac{1}{\sin^{2}\theta }\partial_{\varphi }X \end{array}\right), \end{array}$$

(41) $$\begin{array}{c} {\mathrm{div}}_{r}X_{r}=\frac{1}{\sin\theta }\;\partial_{\theta }\left(X_{r}^{\theta }\sin\theta \right)+\partial_{\varphi }\;X_{r}^{\varphi }. \end{array}$$

In the five-dimensional space, the gradient is given by

(42) $$\begin{array}{c} \mathrm{grad}X=\left(\begin{array}{c} \nabla X\\ {\mathrm{grad}}_{r}X \end{array}\right)\;\text{with }\nabla X=\left(\begin{array}{c} \partial_{x}X\\ \partial_{y}X\\ \partial_{z}X \end{array}\right), \end{array}$$

and the divergence of a five-dimensional vector, **X** = (**X**_*t*_, **X**_*r*_)^*T*^, is

(43) $$\begin{array}{c} \mathrm{div}X=\nabla \cdot X_{t}+{\mathrm{div}}_{r}X_{r}. \end{array}$$

Using these notations, equation (34) can be written in the form of a local conservation law involving the five-dimensional current density, **J**_*C*_ = (*j*_*t*_, *j*_*r*_)^*T*^, as

(44) $$\begin{array}{c} \partial_{t}f+\mathrm{div}J_{C}=0,\\ \text{or }\partial_{t}f+\nabla \cdot j_{t}+{\mathrm{div}}_{r}j_{r}=0, \end{array}$$

with translational current density

(45) $$\begin{array}{c} j_{t}=Vf-D_{t}\nabla f=fV_{\text{s}d}u+f\underset{k}{\sum }\left(\xi_{k}1+\varepsilon_{k}\;Q_{u}\right)\cdot \nabla n_{k}-D_{t}\left(\nabla f+f\beta \nabla U_{t}\right), \end{array}$$

where *U*_*t*_(*r*) = −*F*_ext_ · *r* is the translational potential energy due to the external force *F*_ext_, rotational current density

(46) $$\begin{array}{c} j_{r}=-D_{r}{\text{e}}^{-\beta U_{r}}{\mathrm{grad}}_{r}\left({\text{e}}^{\beta U_{r}}f\right)=f\left(\underset{k}{\sum }\lambda_{k}\nabla n_{k}\right)\cdot {\mathrm{grad}}_{r}u-D_{\text{r}}\left({\mathrm{grad}}_{r}f-f\beta \mu B\cdot {\mathrm{grad}}_{r}u\right), \end{array}$$

and their translational and rotational divergences, ∇·*j*_*t*_ and div_*r*_*j*_*r*_ = −*D*_*r*_*L*_*r*_*f*, where *L*_*r*_ is the operator (37).

### 3.4. Nonequilibrium Thermodynamics of the Active Suspension

Local thermodynamic equilibrium is assumed on scales larger than the size of the colloidal motors where the description by the mean-field equations (33) and (34) is valid and the fluid is at rest. According to this assumption, thermodynamic quantities can be locally expressed in the active suspension in terms of the molecular densities *n*_*k*_(*r*, *t*) and the distribution function *f*(*r*, *u*, *t*). Furthermore, we suppose that the system is isothermal and isobaric and the solution is dilute in the species *A*, *B*, and *C*. The appropriate thermodynamic potential is thus Gibbs' free energy given by the following volume integral of the corresponding density:

(47) $$\begin{array}{c} G=\int d^{3}r\left\{n_{S}\psi_{S}+\underset{k=A,B}{\sum }\left(n_{k}\psi_{k}+n_{k}k_{\text{B}}T\ln\frac{n_{k}}{en_{S}}\right)+\int d^{2}u\left[f\psi_{C}+fk_{\text{B}}T\ln\frac{f}{4\pi en_{S}}+fU_{t}\left(r\right)-f\mu B\cdot u\right]\right\}, \end{array}$$

where the first term is the contribution from the solvent *S* of density *n*_*S*_, the next terms from fuel *k* = *A* and product *k* = *B* dilutely dispersed in the solvent [52], and the last terms from all the orientations *u* of the colloidal motors moving in the mechanical potential energies due to the external force *F*_ext_ and the external torque exerted by the magnetic field *B* on the magnetic dipoles of the colloids. We can thus deduce the following chemical potentials:

(48) $$\begin{array}{c} \mu_{S}=\frac{\delta G}{\delta n_{S}}=\psi_{S}-\frac{k_{\text{B}}T}{n_{S}}\;\left(n_{A}+n_{B}+n_{C}\right), \end{array}$$

(49) $$\begin{array}{c} \mu_{k}=\frac{\delta G}{\delta n_{k}}=\psi_{k}+k_{\text{B}}T\ln\frac{n_{k}}{n_{S}}\;\left(k=A,B\right), \end{array}$$

(50) $$\begin{array}{c} \mu_{C}=\frac{\delta G}{\delta f}=\psi_{C}+k_{\text{B}}T\ln\frac{f}{4\pi n_{S}}+U_{t}\left(r\right)-\mu B\cdot u. \end{array}$$

Here, *ψ*_*k*_ = *μ*_*k*_^0^ + *k*_B_*T*ln(*n*_*S*_/*n*^0^), where *μ*_*k*_^0^ is the standard chemical potential of species *k* and *n*^0^ = 1 mole/liter is the standard concentration. Since the solution is dilute, we have taken the solvent density *n*_S_ to be essentially uniform in space and constant in time.

Next, we use the principles of nonequilibrium thermodynamics in order to express the current densities in terms of the affinities or thermodynamic forces given in Table 1. For the reaction (32), the affinity is given by

(51) $$\begin{array}{c} A_{rxn}=\frac{1}{k_{\text{B}}T}\left(\mu_{A}-\mu_{B}\right)=\ln\frac{k_{+}n_{A}}{k_{-}n_{B}}, \end{array}$$

and the corresponding current density is the rate density *w* introduced in equation (33). At chemical equilibrium, we have *A*_*rxn*_ = 0, *w* = 0, and *k*_+_*n*_*A*,eq_ = *k*_−_*n*_*B*,eq_. In the linear regime close to equilibrium where *δn*_*k*_ = *n*_*k*_ − *n*_*k*,eq_, the chemical affinity (51) can be approximated as

(52) $$\begin{array}{c} A_{rxn}=\frac{\delta n_{A}}{n_{A,\text{eq}}}-\frac{\delta n_{B}}{n_{B,\text{eq}}}=\frac{1}{D_{rxn}}\left(k_{+}\delta n_{A}-k_{-}\delta n_{B}\right), \end{array}$$

where

(53) $$\begin{array}{c} D_{rxn}\equiv \frac{1}{2}{\left(k_{+}n_{A}+k_{-}n_{B}\right)}_{\text{eq}}, \end{array}$$

is the diffusivity of the reaction taking place on the colloidal motors [39, 40]. For the diffusion processes of species *k*, the affinity associated with the current density *j*_*k*_ is given by *A*_*k*_ = −grad(*μ*_*k*_/*k*_B_*T*) in terms of the chemical potential *μ*_*k*_. For molecular species, the gradient is tridimensional in Euclidian space, so that *A*_*k*_ = −∇(*μ*_*k*_/*k*_B_*T*) = −*n*_*k*_^−1^∇*n*_*k*_. For the colloid with chemical potential (50), the affinity is given by the five-dimensional gradient (42) as

(54) $$\begin{array}{c} A_{C}=-\mathrm{grad}\frac{\mu_{C}}{k_{\text{B}}T}=-\frac{1}{f}\left(\begin{array}{c} \nabla f+f\beta \nabla U_{\text{t}}\\ {\mathrm{grad}}_{r}f-f\beta \mu B\cdot {\mathrm{grad}}_{r}u \end{array}\right), \end{array}$$

if the magnetic field *B* is uniform. In this five-dimensional space, the associated current density **J**_*C*_ = (*j*_*t*_, *j*_*r*_)^*T*^ given by equations (45) and (46) can thus be written in the following form:

(55) $$\begin{array}{c} J_{C}=f\left(\begin{array}{c} V_{sd}u\\ 0 \end{array}\right)+f\underset{k=A,B}{\sum }\left(\begin{array}{c} \xi_{k}1+\varepsilon_{k}{\text{Q}}_{u}\\ \lambda_{k}{\mathrm{grad}}_{r}u \end{array}\right)\cdot \nabla n_{k}-f\left(\begin{array}{cc} D_{t}1 & 0\\ 0 & D_{r}1_{r} \end{array}\right)\cdot \mathrm{grad}\frac{\mu_{C}}{k_{\text{B}}T}, \end{array}$$

where 1_*r*_ is the 2 × 2 identity matrix. In this form, we see that the first term is related to the reaction affinity since the self-diffusiophoretic velocity can be written as *V*_*sd*_ = *χD*_*rxn*_*A*_*rxn*_. The next two terms can be related to the affinities of molecular species, and the last term to the affinity of the colloidal species.

According to the Curie principle, there is no coupling between processes with different tensorial characters. However, the Janus particles have a director given by the unit vector *u* and we have adopted a description in terms of the distribution function *f*(*r*, *u*, *t*) for the Janus particles. Consequently, it is possible that a vectorial process such as diffusion may be coupled to a scalar process such as reaction if it is polarized by the unit vector *u*. If we introduce the densities *𝒩*_*C*_ = *f*Δ^2^*u* for Janus particles having their orientation *u* in cells with a size of Δ^2^*u*, along with the associated current densities,

(56) $$\begin{array}{c} J_{C}=J_{C}\Delta^{2}u, \end{array}$$

we may write a general coupling (25) of the following form:

(57) $$\begin{array}{c} \left(\begin{array}{c} w\\ j_{A}\\ j_{B}\\ J_{C} \end{array}\right)=\left(\begin{array}{cccc} L_{rr} & L_{rA} & L_{rB} & L_{rC}\\ L_{Ar} & L_{AA} & L_{AB} & L_{AC}\\ L_{Br} & L_{BA} & L_{BB} & L_{BC}\\ L_{Cr} & L_{CA} & L_{CB} & L_{CC} \end{array}\right)\cdot \left(\begin{array}{c} A_{rxn}\\ -\nabla \frac{\mu_{A}}{k_{\text{B}}T}\\ -\nabla \frac{\mu_{B}}{k_{\text{B}}T}\\ -\mathrm{grad}\frac{\mu_{C}}{k_{\text{B}}T} \end{array}\right), \end{array}$$

up to possible nonlinear contributions that may be required in order for the reaction rate to obey the mass-action law. In equation (57), we have that *L*_*rr*_ is 1 × 1, *L*_*rk*_1 × 3, *L*_*rC*_1 × 5, *L*_*kr*_3 × 1, L_*kl*_3 × 3, *L*_*kC*_3 × 5, *L*_*Cr*_5 × 1, *L*_*Ck*_5 × 3, and *L*_*CC*_5 × 5 (for *k*, *l* = *A*, *B*).

According to Onsager's reciprocal relations (26), the linear response coefficients should obey

(58) $$\begin{array}{c} L_{rk}=L_{kr}^{T},\\ L_{rC}=L_{Cr}^{T},\\ L_{kl}=L_{lk}^{T},\\ L_{CC}=L_{CC}^{T},\\ L_{kC}=L_{Ck}^{T}, \end{array}$$

for *k* = *A*, *B* and where *T* again denotes the transpose.

We assume that the molecular species *A* and *B* undergo Fickian diffusion without cross-diffusion, so that

(59) $$\begin{array}{c} L_{kl}=D_{k}n_{k}\delta_{kl}1, \end{array}$$

and that the reaction rate does not depend on the gradients ∇*n*_*A*_ or ∇*n*_*B*_, whereupon

(60) $$\begin{array}{c} L_{rA}=L_{rB}=0. \end{array}$$

This last assumption consists in neglecting the terms with the coefficient *ϖ* in equation (23), which is usually justified as mentioned in Section 2.

The scalar coefficient associated with the reaction can be identified as

(61) $$\begin{array}{c} L_{rr}=D_{rxn}n_{C}, \end{array}$$

and the linear response coefficients *L*_*Cr*_, *L*_*Ck*_, and *L*_*CC*_ in equation (57) can be determined using the current density (55), as described in Appendix B. As a consequence of Onsager's reciprocal relations, we can conclude that the reaction rate and the current densities should be given by

(62) $$\begin{array}{c} w=D_{rxn}n_{C}A_{rxn}-\chi D_{rxn}\int u\cdot \left(\nabla f+f\beta \nabla U_{t}\right)d^{2}u, \end{array}$$

(63) $$\begin{array}{c} j_{k}=-D_{k}\nabla n_{k}+n_{k}\int \left[\left(\xi_{k}1+\varepsilon_{k}Q_{u}\right)\cdot \left(\nabla f+f\beta \nabla U_{\text{t}}\right)+\lambda_{k}\left({\mathrm{grad}}_{r}u\right)\cdot \left({\mathrm{grad}}_{r}f-f\beta \mu B\cdot {\mathrm{grad}}_{r}u\right)\right]d^{2}u. \end{array}$$

In equation (62), the second term describes the reciprocal effects of diffusiophoresis back onto reaction. The second term in equation (63) is due to cross-diffusion between the molecular and colloidal species due to diffusiophoresis. We see that the linear response coefficients depend on the unit vector *u* in a manner similar to that already shown in Refs. [39, 40].

With respect to standard expressions, the terms involving the integral ∫*d*^2^*u* in equation (63) are required in order to satisfy Onsager's reciprocal relations and for these quantities to be compatible with microreversibility. However, these extra terms can be shown to be negligible, although the reciprocal terms are not negligible in equations (45) and (46). In order to show that the extra terms are negligible, we suppose that the self-diffusiophoretic and diffusiophoretic velocities take the typical value *V*_*sd*_ ~ *V*_*d*_ ~ 10 *μ*m/*s* [60]. According to Ref. [58], the molecular gradients used in experiments of diffusiophoresis are of the order of ‖∇*n*_*k*_‖ ~ 10^5^ mol/m^4^, so that diffusiophoretic parameters have the value *ξ*_*k*_, *ε*_*k*_ ~ 10^−10^ m^5^ s^−1^ mol^−1^. Moreover, we have *λ*_*k*_ ~ *ξ*_*k*_/*R*, but since ‖grad_*r*_*f*‖ ~ *R*‖∇*f*‖, the effect of the coefficients *λ*_*k*_ is again of the same order of magnitude as *ξ*_*k*_ and *ε*_*k*_. Molecular diffusivities typically have the value *D*_*k*_ ~ 10^−9^ m^2^/s, while the translational diffusion coefficient of a micrometric colloidal particle is of the order of *D*_*t*_ ~ 10^−13^ m^2^/s. The molecular concentrations used in experiments on self-diffusiophoresis are about *n*_*k*_ ~ 10^3^ mol/m^3^, while the density of micrometric colloidal particles is approximately *n*_*C*_ ~ 10^18^m^−3^ ~ 10^−6^mol/m^3^, or lower. If we assume that the molecular and colloidal gradients take comparable values ‖∇*n*_*k*_‖/*n*_*k*_ ~ ‖∇*f*‖/*f*, the ratio between the extra term and the standard molecular diffusion term in equation (63) is given by

(64) $$\begin{array}{c} \frac{n_{k}\;\xi_{k}\;\left\|\nabla f\right\|}{D_{k}||\nabla n_{k}||}\sim \frac{\xi_{k}\;f}{D_{k}}\sim 10^{-8}, \end{array}$$

which shows that the second term in equation (63) is negligible. Accordingly, the standard Fickian expressions *j*_*k*_≃−*D*_*k*_∇*n*_*k*_ are very well justified for the molecular current densities. In the presence of colloidal motors, the expressions compatible with microreversibility are nevertheless given by equations (62) and (63). In contrast, the terms associated with the diffusiophoretic parameters in the colloidal current density (55) have effects that are not negligible.

The conclusion from these considerations is that active matter can be described as generalized diffusion-reaction processes in complete compatibility with microreversibility and Onsager's reciprocal relations. In this way, the program of nonequilibrium thermodynamics is complete and application of equation (27) gives the following expression for the thermodynamic entropy production rate density:

(65) $$\begin{array}{c} k_{B}^{-1}\sigma_{s}=D_{rxn}n_{C}A_{rxn}^{2}+\underset{k=A,B}{\sum }D_{k}\frac{{\left(\nabla n_{k}\right)}^{2}}{n_{k}}-2\chi D_{rxn}A_{rxn}\int u\cdot \left(\nabla f+f\beta \nabla U_{t}\right)d^{2}u-2\int \left[\underset{k=A,B}{\sum }\nabla n_{k}\cdot \left(\xi_{k}1+\varepsilon_{k}Q_{u}\right)\right]\cdot \left(\nabla f+f\beta \nabla U_{t}\right)d^{2}u-2\int \left[\left(\underset{k=A,B}{\sum }\lambda_{k}\nabla n_{k}\right)\cdot \left({\mathrm{grad}}_{r}u\right)\right]\cdot \left({\mathrm{grad}}_{r}f-f\beta \mu B\cdot {\mathrm{grad}}_{r}u\right)d^{2}u+D_{t}\int \frac{1}{f}{\left(\nabla f+f\beta \nabla U_{t}\right)}^{2}d^{2}u+D_{r}\int \frac{1}{f}{\left({\mathrm{grad}}_{r}f-f\beta \mu B\cdot {\mathrm{grad}}_{r}u\right)}^{2}d^{2}u\geq 0. \end{array}$$

The second law is satisfied if *D*_*t*_ ≫ *χ*^2^*D*_*rxn*_ > 0, *D*_*k*_*D*_*t*_ ≫ *n*_*C*_*n*_*k*_*ξ*_*k*_^2^ > 0, *D*_*k*_*D*_*t*_ ≫ *n*_*C*_*n*_*k*_*ε*_*k*_^2^ > 0, and *D*_*k*_*D*_*r*_ ≫ *n*_*C*_*n*_*k*_*λ*_*k*_^2^ > 0, which is expected.

The results derived in this section provide the basis for the analysis of collective effects in suspensions of active Janus particles. In Sections 4 and 5, we describe two collective phenomena that emerge from this theoretical framework: the effect of an external force and torque on the reaction rate, and a clustering instability.

## 4. Effect of External Force and Torque

Using a thermodynamic formulation that is consistent with microreversibility, we showed earlier [39–41, 61] how the application of an external force and torque on a single colloidal motor can change the reaction rate on its surface and even lead to a net production of fuel rather than product. Now we show how these considerations can be extended to a suspension of such motors.

### 4.1. Local Evolution Equations

We suppose that the colloidal motors are subjected to an external force *F*_ext_ = *F*_ext_1_*z*_ and an external torque induced by an external magnetic field *B* = *B*1_*z*_ exerted on the magnetic moment *μ* of the colloidal particles, both oriented in the *z*-direction. If *βμB* is large enough, the distribution function is given by

(66) $$\begin{array}{c} f\left(r,u,t\right)=n\left(r,t\right)\frac{\beta \mu B}{4\pi \sinh\beta \mu B}\exp\left(\beta \mu B\cos\theta \right), \end{array}$$

so that *p*(*r*, *t*) = 1_*z*_〈*u*_*z*_〉*n*_C_(*r*, *t*) with 〈*u*_*z*_〉 = coth*βμB* − (*βμB*)^−1^. Moreover, the terms with the coefficients *ξ*_*k*_, *ε*_*k*_, and *λ*_*k*_ are assumed to be negligible in equation (34). If the concentrations are uniform in the *x*- and *y*-directions, the process is ruled by

(67) $$\begin{array}{c} \partial_{t}n_{C}+\partial_{z}\left[\chi \langle u_{z}\rangle \left(k_{+}n_{A}-k_{-}n_{B}\right)n_{C}-D_{t}\left(\partial_{z}n_{C}-\beta F_{\text{ext}}n_{C}\right)\right]=0, \end{array}$$

obtained by integrating equation (34) over the orientation *u*. This equation for *n*_C_ is coupled to equation (33) with the Fickian molecular current densities *j*_*k*_≃−*D*_*k*_∇*n*_*k*_ and the local reaction rate

(68) $$\begin{array}{c} w=\left(k_{+}n_{A}-k_{-}n_{B}\right)n_{C}-\chi \langle u_{z}\rangle D_{rxn}\left(\partial_{z}n_{C}-\beta F_{\text{ext}}n_{C}\right), \end{array}$$

given by equation (62), as predicted by Onsager's reciprocal relations.

### 4.2. Global Evolution Equations

Defining the mean value of the *z*-coordinate for the colloidal motors as

(69) $$\begin{array}{c} \langle z\rangle \equiv \frac{\int zn_{C}d^{3}r}{\int n_{C}d^{3}r}, \end{array}$$

and using equation (67), we obtain the evolution equation,

(70) $$\begin{array}{c} \frac{d\langle z\rangle }{dt}=\chi \langle u_{z}\rangle w_{rxn}+\beta D_{t}F_{\text{ext}}, \end{array}$$

with mean reaction rate,

(71) $$\begin{array}{c} w_{rxn}\equiv k_{+}\frac{\int n_{A}n_{C}d^{3}r}{\int n_{C}d^{3}r}-k_{-}\frac{\int n_{B}n_{C}d^{3}r}{\int n_{C}d^{3}r}. \end{array}$$

Furthermore, integrating equation (33) with *k* = *B* over the position *r* with the local rate (68), we get the total reaction rate

(72) $$\begin{array}{c} \frac{dN_{rxn}}{dt}=\frac{dN_{B}}{dt}=-\frac{dN_{A}}{dt}=N_{C}\left(w_{rxn}+\chi \langle u_{z}\rangle D_{rxn}\beta F_{\text{ext}}\right), \end{array}$$

where *N*_*C*_ = ∫*n*_*C*_*d*^3^*r* is the total number of colloidal motors in the suspension. Equations (70) and (72) have precisely the same structure as for a single colloidal motor. However, one should note that the mean reaction rate in equation (71) contains spatial correlations between the solute and colloid concentration fields. Given the structure of the equations, the results obtained in Refs. [39, 40] also apply here. In particular, there exists a regime where the entire ensemble of colloidal motors is propelled and carries out work against the external force by consuming fuel. In addition, there is also a regime where fuel is synthesized if the external force that opposes motion is sufficiently large to reverse the reaction *A*⟶*B*. The efficiencies of these processes are given by the same expressions as in Refs. [39, 40].

## 5. Clustering Instability and Pattern Formation

The equations of motion developed in Section 3 that describe a dilute suspension of colloidal motors moving in a dilute solution of fuel *A* and product *B* molecular species will be shown in this section to lead to a clustering instability. This instability can be described by the mean-field equations obtained above for the concentrations of the molecular species and the distribution function of the colloidal motors in the absence of an external force and torque (*F*_ext_ = 0 and *B* = 0). A number of other mean-field descriptions that predict instabilities and the formation of various clustering states of collections of diffusiophoretic colloidal particles have appeared in literature [62, 18, 6, 19, 20], and use techniques involving coupled moment equations similar to those adopted in this section.

### 5.1. Molecular Diffusion and Reaction

The equation for the colloidal motors is coupled to the reaction-diffusion equations for the molecular species *A* and *B*, accounting for the fact that the reaction *A*⇄*B* occurs both at the surface of the catalytic hemisphere of the colloids and in the bulk:

(73) $$\begin{array}{c} \partial_{t}n_{k}=D_{k}\nabla^{2}n_{k}+\nu_{k}w_{\text{tot}}, \end{array}$$

where the total reaction rate density is given by

(74) $$\begin{array}{c} w_{\text{tot}}=\left(k_{+}n_{A}-k_{-}n_{B}\right)n_{C}+k_{+2}n_{A}-k_{-2}n_{B}. \end{array}$$

The system is driven out of equilibrium if *k*_+_/*k*_−_ ≠ *k*_+2_/*k*_−2_ [61].

### 5.2. Colloidal Density and Polarization

If the second moment (31) as well as higher moments are assumed to be negligible, the evolution equations for the density of colloidal motors (29) and the polarization (30) are given by

(75) $$\begin{array}{c} \partial_{t}n_{C}+\nabla \cdot \left(n_{C}\underset{k}{\sum }\xi_{k}\nabla n_{k}+V_{sd}p\right)=D_{t}\nabla^{2}n_{C},\; \end{array}$$

(76) $$\begin{array}{c} \partial_{t}p+\nabla \cdot \left(p\underset{k}{\sum }\xi_{k}\nabla n_{k}\right)+\frac{1}{3}\nabla \left(V_{sd}n_{C}\right)+\frac{1}{5}\Delta :\nabla \left(\underset{k}{\sum }\varepsilon_{k}\nabla n_{k}p\right)=D_{t}\nabla^{2}p-2D_{r}p+\frac{2}{3}\;n_{C}\underset{k}{\sum }\lambda_{k}\nabla n_{k}, \end{array}$$

in terms of the fourth-order tensor Δ with the following components: Δ_*ijmn*_ = *δ*_*ij*_*δ*_*mn*_ + *δ*_*im*_*δ*_*jn*_ − (2/3)*δ*_*in*_*δ*_*jm*_.

If *D*_*r*_ is large enough so that 2*D*_*r*_*p* dominates the other terms involving *p* in equation (76), we can neglect these other terms and this equation can be inverted to obtain

(77) $$\begin{array}{c} p≃\frac{1}{6D_{r}}\left[-V_{sd}\nabla n_{C}+n_{C}\underset{k}{\sum }\left(2\lambda_{k}-\zeta_{k}\right)\nabla n_{k}\right], \end{array}$$

under which circumstances the field *p* is driven by the gradients of the colloid and species densities. Substituting this result into equation (75) for the density *n*_C_ of colloidal particles, we find

(78) $$\begin{array}{c} \partial_{t}n_{C}+\nabla \cdot \left\{n_{C}\underset{k}{\sum }\left[\xi_{k}+\frac{V_{sd}}{6D_{r}}\left(2\lambda_{k}-\zeta_{k}\right)\right]\nabla n_{k}-D_{t}^{\left(\text{eff}\right)}\nabla n_{C}\right\}=0, \end{array}$$

with the effective diffusion coefficient

(79) $$\begin{array}{c} D_{t}^{\left(\text{eff}\right)}\equiv D_{t}+\frac{V_{sd}^{2}}{6D_{r}}, \end{array}$$

expressing the enhancement of diffusivity due to the self-diffusiophoretic activity [17].

### 5.3. Coupled Colloidal and Molecular Diffusion-Reaction Equations

In the following, we suppose that the diffusion coefficient is the same for both molecular species: *D* ≡ *D*_*A*_ = *D*_*B*_. Consequently, *n*_0_ = *n*_*A*_ + *n*_*B*_ remains uniform during the time evolution if initially so. Therefore, *n*_*B*_ = *n*_0_ − *n*_*A*_ is known and only *n*_*A*_ needs to be determined. Introducing the notations

(80) $$\begin{array}{c} a\equiv n_{A},\\ c\equiv n_{C}, \end{array}$$

we have the following coupled equations describing the system:

(81) $$\begin{array}{c} \partial_{t}a=D\nabla^{2}a-W_{\text{tot}}, \end{array}$$

(82) $$\begin{array}{c} W_{\text{tot}}=c\left(K\;a-K_{0}\right)+K_{2}a-K_{20}, \end{array}$$

(83) $$\begin{array}{c} \partial_{t}c=\nabla \cdot \left[\left(D_{t}+\tau_{r}V_{sd}^{2}\right)\nabla c-\left(\xi +\sigma V_{sd}\right)c\nabla a\right], \end{array}$$

(84) $$\begin{array}{c} V_{sd}=\zeta a-V_{0}, \end{array}$$

with

(85) $$\begin{array}{c} K\equiv k_{+}+k_{-},\\ K_{0}\equiv k_{-}n_{0},\\ K_{2}\equiv k_{+2}+k_{-2},\\ K_{20}\equiv k_{-2}n_{0},\\ \tau_{r}\equiv {\left(6D_{r}\right)}^{-1},\\ \xi \equiv \xi_{A}-\xi_{B},\\ \lambda \equiv \lambda_{A}-\lambda_{B},\\ \zeta \equiv \zeta_{A}-\zeta_{B}=\varsigma \left(\kappa_{+}^{c}+\kappa_{-}^{c}\right),\\ V_{0}\equiv -\zeta_{B}n_{0}=\varsigma \;\kappa_{-}^{c}\;n_{0},\\ \sigma \equiv \tau_{r}\left(2\lambda -\zeta \right). \end{array}$$

Moreover, consistency with the existence of equilibrium requires that *ζ*/*K* = *V*_0_/*K*_0_ = *ς*/Γ = *χ* is equal to the diffusiophoretic parameter (24) that is the ratio between the self-diffusiophoretic velocity (21) and the leading term of the overall reaction rate (23).

For this system, there exists a uniform nonequilibrium steady state, where *c* keeps its initial uniform value *c*_0_ and the molecular density is also uniform at the value

(86) $$\begin{array}{c} a_{0}=\frac{c_{0}\;K_{0}+K_{20}}{c_{0}\;K+K_{2}}, \end{array}$$

in order to satisfy the stationary condition *W*_tot_ = 0. For this molecular concentration *a* = *a*_0_, we notice that the rate *Ka* − *K*_0_ of the catalytic reaction on the colloids is not vanishing under the nonequilibrium condition *k*_+_/*k*_−_ ≠ *k*_+2_/*k*_−2_.

### 5.4. Pattern Formation

**Figure 2:**
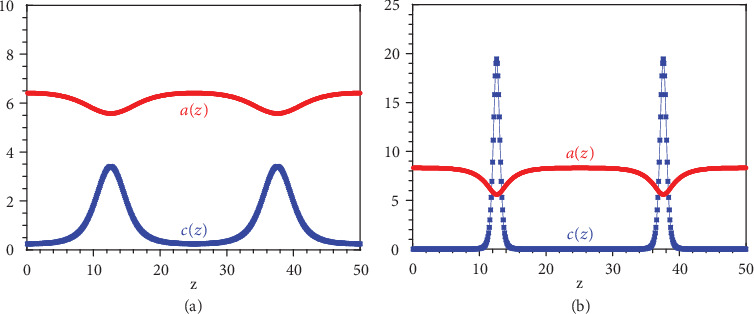
Nonequilibrium steady state of the one-dimensional system for the parameter values (87): (a) with *K*_20_ = 2; (b) with *K*_20_ = 2.5.

To analyze the stability of this homogeneous steady state, for simplicity we consider a one-dimensional system where the fields *a* and *c* only depend on the variable *z*. Accordingly, the gradients ∇ can be replaced by partial derivatives *∂*_*z*_ in equations (81) and (83). The set of equations (81)–(84) is then numerically integrated by spatial discretization over the grid *z* = *i*Δ*z* with *i* = 1, 2, ⋯, *M* with Δ*z* = 0.1 and *M* = 500. The integration is performed with a Runge-Kutta algorithm of varying order 4-5 over a long enough time interval to reach a steady state.  Figure 2 shows numerical results for the parameter values:

(87) $$\begin{array}{c} c_{0}=1,\\ n_{0}=10,\\ D=1,\\ D_{t}=1,\\ \tau_{r}=1,\\ \xi =-3,\\ \sigma =-2,\\ V_{0}=0.5,\\ \zeta =0.1,\\ K=0.2,\\ K_{0}=1,\\ K_{2}=0.3, \end{array}$$

and increasing values of *K*_20_. We observe the formation of clusters of colloidal motors in regions where the fuel *A* is depleted, as expected.

### 5.5. Linear Analysis of the Clustering Instability

The threshold of this clustering instability can be found from a linear stability analysis. Linearizing the equations around the uniform steady state, we find that the perturbations obey

(88) $$\begin{array}{c} \partial_{t}\left(\begin{array}{c} \delta a\\ \delta c \end{array}\right)=\left(\begin{array}{cc} D\partial_{z}^{2}-\tilde{K} & -w\\ -\rho \;\partial_{z}^{2} & D_{\text{t}}^{\left(\text{eff}\right)}\partial_{z}^{2} \end{array}\right)\left(\begin{array}{c} \delta a\\ \delta c \end{array}\right), \end{array}$$

with

(89) $$\begin{array}{c} \tilde{K}\equiv c_{0}K+K_{2},\\ w\equiv Ka_{0}-K_{0},\\ \rho \equiv c_{0}\left(\xi +\sigma V_{sd}\right),\\ D_{t}^{\left(\text{eff}\right)}\equiv D_{t}+\tau_{r}V_{sd}^{2},\\ V_{\text{s}d}\equiv \zeta \;a_{0}-V_{0}. \end{array}$$

Supposing that the perturbations behave as *δa*, *δc* ~ exp(*iqz* + *st*), we obtain the dispersion relations

(90) $$\begin{array}{c} s_{\pm }\left(q\right)=-\frac{1}{2}\left[\tilde{K}+\left(D+D_{t}^{\left(\text{eff}\right)}\right)q^{2}\right]\pm \frac{1}{2}\sqrt{{\left[\tilde{K}+\left(D-D_{t}^{\left(\text{eff}\right)}\right)q^{2}\right]}^{2}-4\rho wq^{2}}. \end{array}$$

**Figure 3:**
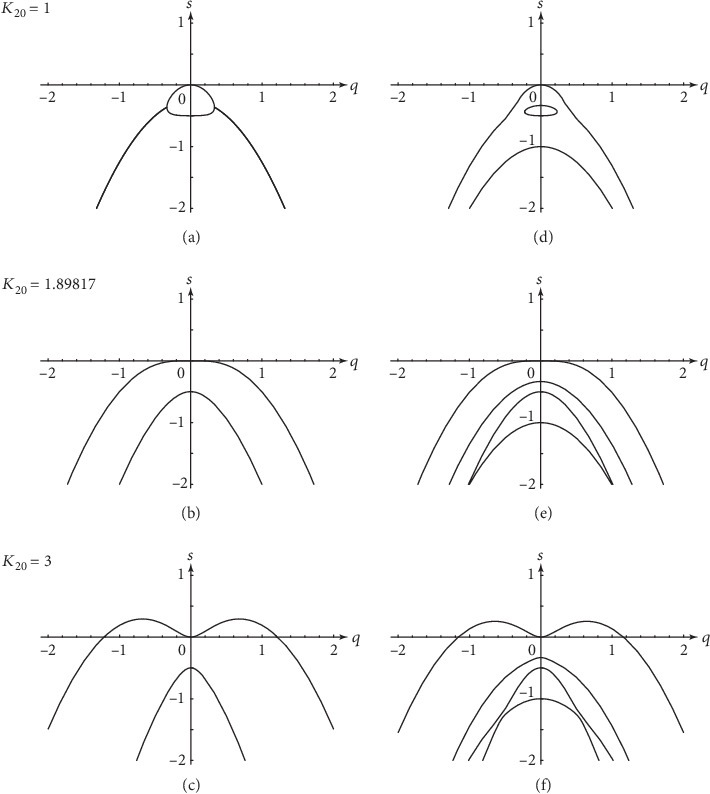
Dispersion relations of linear stability analysis for *K*_20_ = 1, *K*_20_ = 1.89817, and *K*_20_ = 3, respectively, below, at, and beyond the threshold of clustering instability. The dispersion relations are obtained in (a)–(c) with the approximation (90), and in (d)–(f) by truncating equation (C.5) into a 5 × 5 matrix. The other parameter values are given in equation (87) and *ε* = 1.

These dispersion relations are depicted in Figures 3(a)–3(c), respectively, below, at, and beyond the threshold. The leading dispersion relation is associated with the conserved unstable mode of the colloidal motors because *s*_+_(0) = 0. The subleading dispersion relation is associated with the reactive mode of the molecular species because $s_{-}\left(0\right)=-\tilde{K}$. We notice that, since $\tilde{K}>0$, there is no possibility for a Hopf bifurcation to uniform oscillatory behavior, which would be the case if the eigenvalues *s*_±_(0) were complex. We also note that there is no wavelength selection at the level of linear stability analysis in this clustering instability.

Therefore, instability manifests itself if

(91) $$\begin{array}{c} \rho w+\tilde{K}D_{t}^{\left(\text{eff}\right)}<0, \end{array}$$

and the threshold is given by the condition

(92) $$\begin{array}{c} \rho w+\tilde{K}D_{t}^{\left(\text{eff}\right)}=0, \end{array}$$

which leads to the value *K*_20_≃1.89817 for the parameter values (87).

The dispersion relations can also be obtained from the evolution equation (34) for the distribution function. Supposing that *f* = *f*(*z*, *θ*) and *a* = *a*(*z*), we have

(93) $$\begin{array}{c} \partial_{t}f+\partial_{z}\left(V_{dz}f-D_{t}\partial_{z}f\right)=\frac{D_{r}}{\sin\theta }\partial_{\theta }\left(\sin\theta \partial_{\theta }f\right)+2\lambda \cos\theta f\partial_{z}a, \end{array}$$

where

(94) $$\begin{array}{c} V_{dz}=V_{sd}\cos\theta +\left[\xi +\varepsilon \left(\cos^{2}\theta -\frac{1}{3}\right)\right]\partial_{z}a, \end{array}$$

with *ε* ≡ *ε*_*A*_ − *ε*_*B*_. Equation (93) is coupled to the diffusion-reaction equation (81) for the concentration field *a* with *c* = ∫*fd*^2^*u*.

The linear stability analysis can be carried out for equation (93) coupled to equation (81) with the rate (82) in a similar manner to that for equations (81)–(84). This analysis is presented in Appendix C. The dispersion relations can be computed by truncating the infinite matrix (C.5) in order to obtain the eigenvalues as a function of the wave number *q*. The result converges to the dispersion relations shown in Figures 3(d)–3(f) below, at, and beyond the threshold, for the parameter values (87) and *ε* = 1. The convergence occurs faster for the leading dispersion relation than for the subleading ones. For the chosen parameter values, we can see that the approximation where we suppose that the vector field *p* is driven by the gradients (which corresponds to truncating to a 2 × 2 matrix) constitutes a good approximation to describe the instability. Indeed, the leading dispersion relation of Figure 3(b) is already very close to that in Figure 3(e).

The conclusion is that equations (81)–(84) provide a robust description of the clustering instability and of the emerging patterns.

## 6. Conclusion

Autonomous motion is not possible at equilibrium and active matter relies on the presence of nonequilibrium constraints to drive the system out of equilibrium. As a result the theoretical formulations provided by nonequilibrium thermodynamics and statistical mechanics are a natural starting point for the description of such systems.

Many of the active matter systems currently under study involve active agents such as molecular machines or self-propelled colloidal particles with linear dimensions ranging from tens of nanometers to micrometers. The transition from microscopic to macroscopic description for fluids containing active agents of such sizes takes place in the upper range of this scale. Suspensions of active colloidal particles are interesting in this connection since, as described earlier in this paper, the colloidal particles are large compared to the molecules of the medium in which they reside. The dynamics of the suspension can then be described by considering the equations for the positions, velocities, and orientations of the colloidal particles in the medium, or through field equations that describe the densities of these particles.

Nonequilibrium thermodynamics provides a set of principles that these systems must obey. Most important among these is microreversibility that stems from the basic time reversal character of the microscopic dynamics. On the macroscale, this principle manifests itself in Onsager's reciprocal relations that govern what dynamical processes are coupled and how they are described. For example, for single Janus particles propelled by a self-diffusiophoretic mechanism, microreversibility implies the existence of reciprocal effect where the reaction rate depends on an applied external force [39–41, 61].

This paper extended the nonequilibrium thermodynamics formulation to the collective dynamics of ensembles of diffusiophoretic Janus colloids. In particular, we considered Janus colloids driven by both self-diffusiophoresis arising from reactions on the motor catalytic surface as well as motion arising from an external concentration gradient. This latter contribution is essential for the extension of the theory to collective motor dynamics. The resulting formulation is consistent with microreversibility and an expression for the entropy production is provided. From this general formulation of collective dynamics, one can show that if an external force and torque are applied to the system, the overall reaction rate depends on the applied force. In addition, a stability analysis of the equations governing the collective behavior predicts the existence of a clustering instability seen in many experiments of Janus colloids. Such considerations can be extended to ensembles of thermophoretic Janus colloids [63].

## Figures and Tables

**Table 1:** Current densities and corresponding affinities or thermodynamic forces in the active suspension: *w* is the reaction rate density introduced in equation (33) corresponding to the affinity (51), *j*_*A*_ and *j*_*B*_ are the molecular current densities of fuel *A* and product *B*, *j*_*t*_ is the translational current density of colloids given by equation (45), *j*_*r*_ is the rotational current density of colloids given by equation (46). The translational and rotational current densities of colloids form the five-dimensional current density (55) according to **J**_*C*_ = (*j*_*t*_, *j*_*r*_)^*T*^. Similarly, the translational and rotational affinities of colloids form the five-dimensional affinity (54). *β* = (*k*_B_*T*)^−1^ denotes the inverse temperature, *μ*_*k*_ the chemical potentials, ∇ = (*∂*_*x*_, *∂*_*y*_, *∂*_*z*_) the gradient in Euclidean space, and grad_*r*_ the rotational gradient (40). We note that colloidal motors with the given orientation *u* are considered as so many independent species in the free energy (47), which is expressed by equation (56).

Process	Current	Affinity	Dimension
Reaction	*w*	*β*(*μ*_*A*_ − *μ*_*B*_)	1
Molecular diffusion of fuel	*j*_*A*_	−∇(*βμ*_*A*_)	3
Molecular diffusion of product	*j*_*B*_	−∇(*βμ*_*B*_)	3
Translational diffusion of colloids	*j*_*t*_	−∇(*βμ*_*C*_)	3
Rotational diffusion of colloids	*j*_*r*_	−grad_*r*_(*βμ*_*C*_)	2

## A. Force and Torque on a Colloidal Motor

*A.1. Stationary Molecular Concentration Fields*. We suppose that molecular diffusivity is large enough so that the concentration fields adopt a quasistationary profile in the vicinity of every Janus particle. Accordingly, the concentration fields should obey the following equations:

(A.1) $$\begin{array}{c} \nabla^{2}c_{k}=0,\\ D_{k}{\left(\partial_{r}c_{k}\right)}_{R}=-\nu_{k}H^{c}\left(\theta \right){\left(\kappa_{+}^{c}c_{A}-\kappa_{-}^{c}c_{B}\right)}_{R},\\ {\left(\nabla c_{k}\right)}_{\infty }=g_{k}, \end{array}$$

for the two reacting species *k* = *A*, *B*, where *g*_*k*_ is the concentration gradient of species *k* at large distances from the center of the catalytic particle. In Ref. [40], we considered the special case where *g*_*k*_ = 0, so that the concentration fields are uniform at large distances. The upper hemisphere is catalytic, while the lower one is noncatalytic. The axis of the Janus particle is oriented from the noncatalytic towards the catalytic hemisphere and taken along the *z*-axis in the frame of the particle.

We introduce the fields

(A.2) $$\begin{array}{c} \Phi \equiv \ell \left(D_{A}c_{A}+D_{B}c_{B}\right), \end{array}$$

(A.3) $$\begin{array}{c} \Psi \equiv \ell^{2}\left(\kappa_{+}^{c}c_{A}-\kappa_{-}^{c}c_{B}\right), \end{array}$$

in terms of the characteristic length of the reaction

(A.4) $$\begin{array}{c} \ell \equiv {\left(\frac{\kappa_{+}^{c}}{D_{A}}+\frac{\kappa_{-}^{c}}{D_{B}}\right)}^{-1}. \end{array}$$

The fields *Φ* and Ψ have the units of sec^−1^, and the concentration fields are recovered from them by

(A.5) $$\begin{array}{c} c_{A}=\frac{1}{D_{A}}\left(\frac{\kappa_{-}^{c}}{D_{B}}\Phi +\frac{1}{\ell }\Psi \right),\\ c_{B}=\frac{1}{D_{B}}\left(\frac{\kappa_{+}^{c}}{D_{A}}\Phi -\frac{1}{\ell }\Psi \right). \end{array}$$

Similar expressions hold for the concentration gradients at large distances: *g*_*A*_, *g*_*B*_, *g*_*Φ*_, and *g*_Ψ_.

The fields (A.2) and (A.3) obey the equations

(A.6) $$\begin{array}{c} \nabla^{2}\Phi =0,\\ {\left(\partial_{r}\Phi \right)}_{R}=0,\\ {\left(\nabla \Phi \right)}_{\infty }=g_{\Phi },\\ \nabla^{2}\Psi =0,\\ {\left(\partial_{r}\Psi \right)}_{R}=\ell^{-1}H^{c}\left(\theta \right){\left(\Psi \right)}_{R},\\ {\left(\nabla \Psi \right)}_{\infty }=g_{\Psi }, \end{array}$$

where *H*^c^(*θ*) is the Heaviside function of the catalytic hemisphere.

The solution of the equations for *Φ* is given by

(A.7) $$\begin{array}{c} \Phi =\Phi_{g}=\Phi_{0}+g_{\Phi }\cdot r\left[1+\frac{1}{2}{\left(\frac{R}{r}\right)}^{3}\right], \end{array}$$

(A.8) $$\begin{array}{c} \Phi_{0}=\ell \left(D_{A}{\bar{c}}_{A}+D_{B}{\bar{c}}_{B}\right), \end{array}$$

which obeys reflective boundary conditions on the sphere *r* = *R* and represents a gradient at large distances. Defining Da ≡ *R*/*ℓ* to be the dimensionless Damköhler number of the reaction on the spherical catalytic particle, the field Ψ can be decomposed as

(A.9) $$\begin{array}{c} \Psi =\Psi_{g}-\text{Da}\Psi_{0}F, \end{array}$$

in terms of a field similar to equation (A.8)

(A.10) $$\begin{array}{c} \Psi_{g}=\Psi_{0}+g_{\Psi }\cdot r\left[1+\frac{1}{2}{\left(\frac{R}{r}\right)}^{3}\right],\\ \Psi_{0}\equiv \ell^{2}\left(\kappa_{+}^{c}{\bar{c}}_{A}-\kappa_{-}^{c}\;{\bar{c}}_{B}\right), \end{array}$$

and another field *F* obeying

(A.11) $$\begin{array}{c} \nabla^{2}F=0,\\ R{\left(\partial_{r}F\right)}_{R}=H^{c}\left(\theta \right)\left[\text{Da}{\left(F\right)}_{R}-\frac{{\left(\Psi_{g}\right)}_{R}}{\Psi_{0}}\right],\\ {\left(\nabla F\right)}_{\infty }=0. \end{array}$$

In the equations above, the concentrations ${\bar{c}}_{k}$ may be considered as their extrapolations to the center of the Janus particle or the mean concentrations at that position in a dilute suspension of Janus particles. Similarly, *g*_*k*_ may also be considered as the mean gradients of concentrations at the location of the Janus particle in a dilute suspension.

*A.2. Calculation of the Diffusiophoretic Velocities.* The diffusiophoretic force (14) and torque (15) are thus given by the following expressions:

(A.12) $$\begin{array}{c} F_{d}=\frac{6\pi \eta R}{1+\left(3b/R\right)}\;\left[\frac{3}{2}\;{\overset{¯}{b_{A}1_{⊥}}}^{s}\cdot g_{A}+\frac{3}{2}\;{\overset{¯}{b_{B}1_{⊥}}}^{s}\cdot g_{B}+R\left(\kappa_{+}^{c}\;{\bar{c}}_{A}-\kappa_{-}^{c}\;{\bar{c}}_{B}\right){\overset{¯}{\left(\frac{b_{B}}{D_{B}}-\frac{b_{A}}{D_{A}}\right)1_{⊥}\cdot \nabla F}}^{s}\right], \end{array}$$

and

(A.13) $$\begin{array}{c} T_{d}=\frac{12\pi \eta R}{1+\left(3b/R\right)}\;\left[\frac{3R}{2}\;{\overset{¯}{b_{A}n}}^{s}\times g_{A}+\frac{3R}{2}\;{\overset{¯}{b_{B}n}}^{s}\times g_{B}+R\left(\kappa_{+}^{c}\;{\bar{c}}_{A}-\kappa_{-}^{c}{\bar{c}}_{B}\right){\overset{¯}{\left(\frac{b_{B}}{D_{B}}-\frac{b_{A}}{D_{A}}\right)r\times \nabla F}}^{s}\right]. \end{array}$$

Next, the field *F* can be expanded in Legendre polynomials. Since it obeys Laplace's equation and is vanishing at large distances, we find that

(A.14) $$\begin{array}{c} F\left(r,\theta ,\varphi \right)=\overset{\infty }{\underset{l=0}{\sum }}a_{l}P_{l}\left(cos\theta \right){\left(\frac{R}{r}\right)}^{l+1}+\frac{3R}{2\Psi_{0}}g_{\Psi z}\overset{\infty }{\underset{l=0}{\sum }}b_{l}P_{l}\left(cos\theta \right){\left(\frac{R}{r}\right)}^{l+1}+\frac{3R}{2\Psi_{0}}\sqrt{2}\left(g_{\Psi x}cos\varphi +g_{\Psi y}sin\varphi \right)\times \overset{\infty }{\underset{l=1}{\sum }}c_{l}\frac{P_{l}^{1}\left(cos\theta \right)}{\sqrt{l\left(l+1\right)}}{\left(\frac{R}{r}\right)}^{l+1}, \end{array}$$

with

(A.15) $$\begin{array}{c} a_{l}\equiv {\left(M^{-1}\cdot A\right)}_{l}, \end{array}$$

(A.16) $$\begin{array}{c} b_{l}\equiv {\left(M^{-1}\cdot B\right)}_{l}, \end{array}$$

(A.17) $$\begin{array}{c} c_{l}\equiv {\left(N^{-1}\cdot C\right)}_{l}, \end{array}$$

(A.18) $$\begin{array}{c} A_{l}\equiv \int_{0}^{1}d\xi P_{l}\left(\xi \right), \end{array}$$

(A.19) $$\begin{array}{c} B_{l}\equiv \int_{0}^{1}d\xi P_{1}\left(\xi \right)P_{l}\left(\xi \right), \end{array}$$

(A.20) $$\begin{array}{c} C_{l}\equiv \int_{0}^{1}d\xi \frac{P_{1}^{1}\left(\xi \right)}{\sqrt{2}}\frac{P_{l}^{1}\left(\xi \right)}{\sqrt{l\left(l+1\right)}}, \end{array}$$

(A.21) $$\begin{array}{c} M_{ll^{\prime }}\equiv 2\frac{l+1}{2l+1}\delta_{ll^{\prime }}+\text{Da}\int_{0}^{1}d\xi P_{l}\left(\xi \right)P_{l^{\prime }}\left(\xi \right), \end{array}$$

(A.22) $$\begin{array}{c} N_{ll^{\prime }}\equiv 2\frac{l+1}{2l+1}\delta_{ll^{\prime }}+\text{Da}\int_{0}^{1}\;d\xi \frac{P_{l}^{1}\left(\xi \right)}{\sqrt{l\left(l+1\right)}}\frac{P_{l^{\prime }}^{1}\left(\xi \right)}{\sqrt{l^{\prime }\left(l^{\prime }+1\right)}}. \end{array}$$

First, we calculate the force (A.12) in order to obtain the corresponding velocity *V*_*d*_. We have that

(A.23) $$\begin{array}{c} {\overset{¯}{b_{k}nn}}^{s}=\frac{1}{6}\;\left(b_{k}^{c}+b_{k}^{n}\right)1,\\ {\overset{¯}{b_{k}1_{⊥}}}^{s}=\frac{1}{3}\;\left(b_{k}^{c}+b_{k}^{n}\right)1, \end{array}$$

and, furthermore,

(A.24) $$\begin{array}{c} 1_{x}\cdot {\overset{¯}{b_{k}1_{⊥}\cdot \nabla F}}^{s}=-\frac{1}{2}\left(\gamma^{c}b_{k}^{c}+\gamma^{n}b_{k}^{n}\right)\frac{g_{\Psi x}}{\Psi_{0}},\\ 1_{y}\cdot {\overset{¯}{b_{k}1_{⊥}\cdot \nabla F}}^{s}=-\frac{1}{2}\left(\gamma^{c}b_{k}^{c}+\gamma^{n}b_{k}^{n}\right)\frac{g_{\Psi y}}{\Psi_{0}},\\ 1_{z}\cdot {\overset{¯}{b_{k}1_{⊥}\cdot \nabla F}}^{s}=-\frac{1}{2R}\left(\alpha^{c}b_{k}^{c}+\alpha^{n}b_{k}^{n}\right)-\frac{1}{2}\left(\beta^{c}b_{k}^{c}+\beta^{n}b_{k}^{n}\right)\frac{g_{\Psi z}}{\Psi_{0}}, \end{array}$$

in terms of the integrals

(A.25) $$\begin{array}{c} \alpha^{h}\equiv \int d\theta H^{h}\left(\theta \right)\sin^{2}\theta \frac{dA}{d\theta },\\ \beta^{h}\equiv \frac{3}{2}\int d\theta H^{h}\left(\theta \right)\sin^{2}\theta \frac{dB}{d\theta }d,\\ \gamma^{h}\equiv -\frac{3\sqrt{2}}{4}\int d\theta H^{h}\left(\theta \right)\left(\sin\theta \cos\theta \frac{dC}{d\theta }+C\right),\; \end{array}$$

where

(A.26) $$\begin{array}{c} A\equiv \overset{\infty }{\underset{l=0}{\sum }}a_{l}P_{l}\left(\cos\theta \right),\\ B\equiv \overset{\infty }{\underset{l=0}{\sum }}b_{l}P_{l}\left(\cos\theta \right),\\ C\equiv \overset{\infty }{\underset{l=1}{\sum }}c_{l}\frac{P_{l}^{1}\left(\cos\theta \right)}{\sqrt{l\left(l+1\right)}}. \end{array}$$

Then, we calculate the torque (A.13) to get the corresponding angular velocity **Ω**_*d*_. We have that

(A.27) $$\begin{array}{c} {\overset{¯}{b_{k}n}}^{s}=\frac{1}{4}\left(b_{k}^{c}-b_{k}^{n}\right)u,\\ 1_{x}\cdot {\overset{¯}{b_{k}r\times \nabla F}}^{s}=\frac{R}{2}\left(\delta^{c}b_{k}^{c}+\delta^{n}b_{k}^{n}\right)\frac{g_{\Psi y}}{\Psi_{0}},\\ 1_{y}\cdot {\overset{¯}{b_{k}r\times \nabla F}}^{s}=-\frac{R}{2}\left(\delta^{c}b_{k}^{c}+\delta^{n}b_{k}^{n}\right)\frac{g_{\Psi x}}{\Psi_{0}},\\ 1_{z}\cdot {\overset{¯}{b_{k}r\times \nabla F}}^{s}=0, \end{array}$$

with

(A.28) $$\begin{array}{c} \delta^{h}\equiv -\frac{3\sqrt{2}}{4}\int d\theta H^{h}\left(\theta \right)\left(\sin\theta \frac{dC}{d\theta }+\cos\theta C\right), \end{array}$$

so that

(A.29) $$\begin{array}{c} {\overset{¯}{b_{k}r\times \nabla F}}^{s}=\frac{R}{2\Psi_{0}}\left(\delta^{c}b_{k}^{c}+\delta^{n}b_{k}^{n}\right)u\times g_{\Psi }. \end{array}$$

With the following coefficients associated with diffusiophoresis,

(A.30) $$\begin{array}{c} Y^{h}\equiv \frac{b_{A}^{h}}{D_{A}}-\frac{b_{B}^{h}}{D_{B}}\;\text{for }h=c,n, \end{array}$$

the diffusiophoretic linear and angular velocities can be expressed as

(A.31) $$\begin{array}{c} V_{d}=\frac{F_{d}}{\gamma_{t}}=\frac{1}{1+\left(2b/R\right)}\left\{\frac{1}{2}\left(\alpha^{c}Y^{c}+\alpha^{n}Y^{n}\right)\left(\kappa_{+}^{c}\;{\bar{c}}_{A}-\kappa_{-}^{c}\;{\bar{c}}_{B}\right)u+\frac{1}{2}\left(b_{A}^{c}+b_{A}^{n}\right)g_{A}+\frac{1}{2}\left(b_{B}^{c}+b_{B}^{n}\right)g_{B}+\frac{R}{2}\left(\gamma^{c}Y^{c}+\gamma^{n}Y^{n}\right)\left(\kappa_{+}^{c}g_{A}-\kappa_{-}^{c}g_{B}\right)+\frac{R}{2}\left[\left(\beta^{c}-\gamma^{c}\right)Y^{c}+\left(\beta^{n}-\gamma^{n}\right)Y^{n}\right]\left(\kappa_{+}^{c}\;g_{A}-\kappa_{-}^{c}g_{B}\right)\cdot uu\right\}, \end{array}$$

(A.32) $$\begin{array}{c} \Omega_{d}=\frac{T_{d}}{\gamma_{r}}=\frac{9}{16R}\left[\left(b_{A}^{c}-b_{A}^{n}\right)u\times g_{A}+\left(b_{B}^{c}-b_{B}^{n}\right)u\times g_{B}\right]+\frac{3}{4}\left(\delta^{c}Y^{c}+\delta^{n}Y^{n}\right)u\times \left(\kappa_{+}^{c}g_{A}-\kappa_{-}^{c}g_{B}\right). \end{array}$$

By defining the parameters,

(A.33) $$\begin{array}{c} \zeta_{A}=\varsigma \kappa_{+}^{c}, \end{array}$$

(A.34) $$\begin{array}{c} \varsigma =\frac{1}{2}\frac{\sum_{h}\;\alpha^{h}Y^{h}}{1+\left(2b/R\right)}, \end{array}$$

(A.35) $$\begin{array}{c} \xi_{A}=\frac{1}{2}\frac{\sum_{h}\;b_{A}^{h}}{1+\left(2b/R\right)}+\Xi \kappa_{+}^{c}, \end{array}$$

(A.36) $$\begin{array}{c} \Xi =\frac{R}{6}\frac{\sum_{h}\;\left(\beta^{h}+2\gamma^{h}\right)Y^{h}}{1+\left(2b/R\right)}, \end{array}$$

(A.37) $$\begin{array}{c} \varepsilon_{A}=E\kappa_{+}^{c}, \end{array}$$

(A.38) $$\begin{array}{c} E=\frac{R}{2}\frac{\sum_{h}\;\left(\beta^{h}-\gamma^{h}\right)Y^{h}}{1+\left(2b/R\right)}, \end{array}$$

(A.39) $$\begin{array}{c} \lambda_{A}=\frac{9\left(b_{A}^{c}-b_{A}^{n}\right)}{16R}+\Lambda \kappa_{+}^{c}, \end{array}$$

(A.40) $$\begin{array}{c} \Lambda =\frac{3}{4}\underset{h}{\sum }\delta^{h}Y^{h}, \end{array}$$

the linear and angular velocities in equations (A.31) and (A.32) can be written in the forms given in equations (18) and (19) in the main text.

*A.3. Calculation of the Overall Reaction Rate.* Moreover, we can also calculate the overall reaction rate (16). According to the boundary condition

(A.41) $$\begin{array}{c} D_{A}{\left(\partial_{r}c_{A}\right)}_{R}=H^{c}\left(\theta \right){\left(\kappa_{+}^{c}c_{A}-\kappa_{-}^{c}c_{B}\right)}_{R}, \end{array}$$

the reaction rate on the Janus particle is equivalently given by

(A.42) $$\begin{array}{c} W_{rxn}=\int_{r=R}\;D_{A}{\left(\partial_{r}c_{A}\right)}_{R}d\Sigma , \end{array}$$

with *dΣ* = *R*^2^*d*cos*θdφ*. The concentration field *c*_A_ is again decomposed in terms of *Φ* and Ψ. The contributions from the terms *Φ* = *Φ*_*g*_ and Ψ_*g*_ are vanishing, so that there remains

(A.43) $$\begin{array}{c} W_{rxn}=-\frac{\text{Da}}{\ell }\Psi_{0}\int_{r=R}\;{\left(\partial_{r}F\right)}_{R}d\Sigma . \end{array}$$

Using the expansion (A.14), we obtain the overall reaction rate (23) with the rate constants

(A.44) $$\begin{array}{c} k_{\pm }=4\pi R^{2}a_{0}\kappa_{\pm }, \end{array}$$

and the parameter

(A.45) $$\begin{array}{c} \varpi =\frac{3Rb_{0}}{2a_{0}}, \end{array}$$

where the coefficients *a*_0_ and *b*_0_ are given by equations (A.15) and (A.16) with *l* = 0.

## B. Determination of Onsager's Linear Response Coefficients

*B.1. Rotational Gradient and Divergence.* In Euclidean space, the contravariant *a*^*i*^ and covariant *a*_*i*_ components of a vector *a* ∈ ℝ^3^ coincide: *a*_*i*_ = *a*^*i*^. However, in spherical coordinates, the contravariant *a*_*r*_^*i*^ and covariant *a*_*ri*_ components of a rotational vector *a*_*r*_ ∈ ℝ^2^ are related to each other according to *a*_*ri*_ = ∑_*j*=*θ*,*φ*_ *g*_*ij*_*a*_*r*_^*j*^ in terms of the metric (39). Therefore, the scalar product between two rotational vectors *a*_*r*_, *b*_*r*_ ∈ ℝ^2^ has the following equivalent forms, *a*_*r*_ · *b*_*r*_ = ∑_*i*=*θ*,*φ*_ *a*_*ri*_*b*_*r*_^*i*^ = ∑_*i*=*θ*,*φ*_ *a*_*r*_^*i*^*b*_*ri*_. The inverse of the metric (39) associated with the spherical coordinates is given by

(B.1) $$\begin{array}{c} \left(g^{ij}\right)={\left(g_{ij}\right)}^{-1}=\left(\begin{array}{cc} 1 & 0\\ 0 & \frac{1}{\sin^{2}\theta } \end{array}\right), \end{array}$$

and its determinant by

(B.2) $$\begin{array}{c} g=\det\left(g_{ij}\right)=\sin^{2}\theta . \end{array}$$

Hence, the element of angular integration can be written as $d^{2}u=\sqrt{g}d^{2}q=\sin\theta d\theta d\varphi$. Using contravariant components, the gradient of some function *X* is given by [59]

(B.3) $$\begin{array}{c} {\left({\mathrm{grad}}_{r}X\right)}^{i}=\underset{j}{\sum }\;g^{ij}\frac{\partial X}{\partial q^{j}}, \end{array}$$

and the divergence of some vector **X**_*r*_ by

(B.4) $$\begin{array}{c} {\mathrm{div}}_{r}X_{r}=\underset{i}{\sum }\;\frac{1}{\sqrt{g}}\frac{\partial }{\partial q^{i}}\left(X_{r}^{i}\sqrt{g}\right), \end{array}$$

which leads to equations (40) and (41) with the metric (39) of spherical coordinates.

*B.2. Consequences of Onsager's Principle.* Using the chemical potentials (49) and (50) and the assumptions (59) and (60), equation (57) becomes

(B.5) $$\begin{array}{c} \left(\begin{array}{c} w\\ j_{A}\\ j_{B}\\ J_{C} \end{array}\right)=\left(\begin{array}{cccc} L_{rr} & 0 & 0 & L_{rC}\\ 0 & L_{AA} & 0 & L_{AC}\\ 0 & 0 & L_{BB} & L_{BC}\\ L_{Cr} & L_{CA} & L_{CB} & L_{CC} \end{array}\right)\cdot \left(\begin{array}{c} A_{rxn}\\ -\frac{\nabla n_{A}}{n_{A}}\\ -\frac{\nabla n_{B}}{n_{B}}\\ -\mathrm{grad}\frac{\mu_{C}}{k_{\text{B}}T} \end{array}\right), \end{array}$$

which implies that

(B.6) $$\begin{array}{c} w=L_{rr}A_{rxn}-\underset{u}{\sum }\;L_{rC}\cdot \mathrm{grad}\frac{\mu_{C}}{k_{\text{B}}T}, \end{array}$$

(B.7) $$\begin{array}{c} j_{A}=-L_{AA}\cdot \frac{\nabla n_{A}}{n_{A}}-\underset{u}{\sum }\;L_{AC}\cdot \mathrm{grad}\frac{\mu_{C}}{k_{\text{B}}T}, \end{array}$$

(B.8) $$\begin{array}{c} j_{B}=-L_{BB}\cdot \frac{\nabla n_{B}}{n_{B}}-\underset{u}{\sum }\;L_{BC}\cdot \mathrm{grad}\frac{\mu_{C}}{k_{\text{B}}T}, \end{array}$$

(B.9) $$\begin{array}{c} J_{C}=L_{Cr}A_{rxn}-L_{CA}\cdot \frac{\nabla n_{A}}{n_{A}}-L_{CB}\cdot \frac{\nabla n_{B}}{n_{B}}-\underset{u}{\sum }\;L_{CC}\cdot \mathrm{grad}\frac{\mu_{C}}{k_{\text{B}}T}, \end{array}$$

where the sum extends over the different groups Δ^2^*u* of colloidal motors and

(B.10) $$\begin{array}{c} \mathrm{grad}\frac{\mu_{C}}{k_{\text{B}}T}=\frac{1}{f}\left(\begin{array}{c} \nabla f+f\beta \nabla U_{t}\\ \partial_{\theta }f-f\beta \mu B\cdot \partial_{\theta }u\\ \frac{1}{\sin^{2}\theta }\partial_{\varphi }f-f\frac{\beta \mu }{\sin^{2}\theta }B\cdot \partial_{\varphi }u \end{array}\right). \end{array}$$

Using the expression (55) of the five-dimensional colloidal current density and comparing with the expression (B.9), we find that the linear response coefficients are given by

(B.11) $$\begin{array}{c} L_{Cr}=f\left(\begin{array}{c} \chi \;D_{rxn}\;u\\ 0 \end{array}\right)\Delta^{2}u\delta_{uu\prime },\\ L_{CA}=-fn_{A}\left(\begin{array}{c} \xi_{A}1+\varepsilon_{A}Q_{u}\\ \lambda_{A}{\mathrm{grad}}_{r}u \end{array}\right)\Delta^{2}u\delta_{uu\prime },\\ L_{CB}=-fn_{B}\left(\begin{array}{c} \xi_{B}1+\varepsilon_{B}Q_{u}\\ \lambda_{B}{\mathrm{grad}}_{r}u \end{array}\right)\Delta^{2}u\delta_{uu\prime },\\ L_{CC}=f\left(\begin{array}{cc} D_{t}1 & 0\\ 0 & D_{r}1_{r} \end{array}\right)\Delta^{2}u\delta_{uu\prime }. \end{array}$$

Consequently, we obtain equations (62) and (63).

Remark 1 . Interestingly, the assumption (60), according to which the reaction rate does not depend on the gradients of molecular densities, can be relaxed by taking instead

(B.12) $$\begin{array}{c} L_{rA}=-\varpi k_{+}n_{A}p,\\ L_{rB}=+\varpi k_{-}n_{B}p, \end{array}$$

with the polarization (30). Cross-diffusion between the molecular species *A* and *B* may also be included with the coefficients

(B.13) $$\begin{array}{c} L_{AB}=L_{BA}=Cn_{A}n_{B}1. \end{array}$$

In this general case where the matrix of linear response coefficients in equation (57) is complete, the reaction rate and the molecular current densities are instead given by

(B.14) $$\begin{array}{c} w=\left(k_{+}n_{A}-k_{-}n_{B}\right)n_{C}+\varpi \left(k_{+}\nabla n_{A}-k_{-}\nabla n_{B}\right)\cdot p-\chi D_{rxn}\int u\cdot \left(\nabla f+f\beta \nabla U_{t}\right)d^{2}u, \end{array}$$

(B.15) $$\begin{array}{c} j_{A}=-D_{A}\nabla n_{A}-Cn_{A}\nabla n_{B}-\varpi k_{+}n_{A}A_{rxn}p+n_{A}\int \;\left[\left(\xi_{A}1+\varepsilon_{A}Q_{u}\right)\cdot \left(\nabla f+f\beta \nabla U_{t}\right)+\lambda_{A}\left({\mathrm{grad}}_{r}u\right)\cdot \left({\mathrm{grad}}_{r}f-f\beta \mu B\cdot {\mathrm{grad}}_{r}u\right)\right]d^{2}u, \end{array}$$

(B.16) $$\begin{array}{c} j_{B}=-D_{B}\nabla n_{B}-Cn_{B}\nabla n_{A}+\varpi k_{-}n_{B}A_{rxn}p+n_{B}\int \;\left[\left(\xi_{B}\;1+\varepsilon_{B}\;Q_{u}\right)\cdot \left(\nabla f+f\beta \nabla U_{t}\right)+\lambda_{B}\left({\mathrm{grad}}_{r}u\right)\cdot \left({\mathrm{grad}}_{r}f-f\beta \mu B\cdot {\mathrm{grad}}_{r}u\right)\right]d^{2}u. \end{array}$$

Neglecting the last term in the expression (B.14), we recover a form compatible with the reaction rate (23) obtained in Appendix A by direct calculation using the molecular concentration profiles around a single motor. The scheme has great generality.

## C. Linear Stability Analysis Using the Colloidal Distribution Function

Linearizing equation (93) for *f* around a uniform steady state *f*_0_ = *c*_0_/(4*π*), we get

(C.1) $$\begin{array}{c} \partial_{t}\delta f=D_{t}\partial_{z}^{2}\delta f+\frac{D_{r}}{\sin\theta }\partial_{\theta }\left(\sin\theta \partial_{\theta }\delta f\right)-V_{sd}\cos\theta \partial_{z}\delta f-f\left[\xi +\varepsilon \left(\cos^{2}\theta -\frac{1}{3}\right)\right]\partial_{z}^{2}\delta a+\left(2\lambda -\zeta \right)f\cos\theta \partial_{z}\delta a, \end{array}$$

where *δf* = *f* − *f*_0_, and *δa* is ruled by the first line of matricial equation (88). These linear equations can be solved by expanding *δf* in series of Legendre polynomials as

(C.2) $$\begin{array}{c} \delta f=\frac{1}{4\pi }\overset{\infty }{\underset{l=0}{\sum }}\;\delta c_{l}P_{l}\left(\cos\theta \right). \end{array}$$

Supposing that *δf*, *δa* ~ exp(*iqz*), we find the following coupled equations

(C.3) $$\begin{array}{c} \frac{d\delta c_{l}}{dt}=-\left[D_{t}q^{2}+l\left(l+1\right)D_{r}\right]\delta c_{l}-iqV_{sd}\left(\frac{l}{2l+1}\delta c_{l-1}+\frac{l+1}{2l+3}\delta c_{l+1}\right)+c_{0}\left[q^{2}\left(\xi \delta_{l,0}+\frac{2}{3}\varepsilon \delta_{l,2}\right)+iq\left(2\lambda -\zeta \right)\delta_{l,1}\right]\delta a, \end{array}$$

for *l* = 0, 1, 2, ⋯, and

(C.4) $$\begin{array}{c} \frac{d\delta a}{dt}=-\left(Dq^{2}+\tilde{K}\right)\delta a-w\delta c_{0}. \end{array}$$

In matrix form, we have

(C.5) $$\begin{array}{c} \frac{d}{dt}\left(\begin{array}{c} \delta a\\ \delta c_{0}\\ \delta c_{1}\\ \delta c_{2}\\ \delta c_{3}\\ \delta c_{4}\\ \delta c_{5}\\ \vdots \end{array}\right)=\left(\begin{array}{ccccccc} -Dq^{2}-\tilde{K} & -w & 0 & 0 & 0 & 0 & \cdots \\ c_{0}\xi q^{2} & -D_{t}q^{2} & -i\frac{V_{sd}}{3}q & 0 & 0 & 0 & \cdots \\ ic_{0}\left(2\lambda -\zeta \right)q & -iV_{sd}q & -D_{t}q^{2}-2D_{r} & -i\frac{2V_{sd}}{5}q & 0 & 0 & \cdots \\ c_{0}\frac{2\varepsilon }{3}q^{2} & 0 & -i\frac{2V_{sd}}{3}q & -D_{t}q^{2}-6D_{r} & -i\frac{3V_{sd}}{7}q & 0 & \cdots \\ 0 & 0 & 0 & -i\frac{3V_{sd}}{5}q & -D_{t}q^{2}-12D_{r} & -i\frac{4V_{sd}}{9}q & \cdots \\ 0 & 0 & 0 & 0 & -i\frac{4V_{sd}}{7}q & -D_{t}q^{2}-20D_{r} & \cdots \\ \vdots & \vdots & \vdots & \vdots & \vdots & \vdots & \ddots \end{array}\right)\left(\begin{array}{c} \delta a\\ \delta c_{0}\\ \delta c_{1}\\ \delta c_{2}\\ \delta c_{3}\\ \delta c_{4}\\ \delta c_{5}\\ \vdots \end{array}\right), \end{array}$$

which can be solved by truncation to obtain the dispersion relations shown in Figures 3(d)–3(f). If the wave number is vanishing (*q* = 0), the matrix in equation (C.5) has the following successive eigenvalues: $s_{-}\left(0\right)=-\tilde{K}$ for reaction, *s*_+_(0) = 0 for translational diffusion, and *s*_*l*_(0) = −*l*(*l* + 1)*D*_*r*_ with *l* = 1, 2, 3, ⋯ for rotational diffusion. In Figure 3(b), they appear in the order $s_{+}\left(0\right)=0>s_{1}\left(0\right)=-2D_{r}>s_{-}\left(0\right)=-\tilde{K}>s_{2}\left(0\right)=-6D_{r}>\cdots$ for the parameter values (87). We note that all the dispersion relations satisfy the property *∂*_*q*_*s*(0) = 0. At the threshold of clustering instability, the leading dispersion relation *s*_+_(*q*) should moreover satisfy the condition *∂*_*q*_^2^*s*_+_(0) = 0, which also gives equation (92), thus confirming the validity of the approximation (88) to determine the threshold.
